# Validating 8 Area-Based Measures of Social Risk for Predicting Health and Mortality

**DOI:** 10.1001/jamahealthforum.2025.2669

**Published:** 2025-08-15

**Authors:** Aubrey Limburg, David H. Rehkopf, Nicole Gladish, Robert L. Phillips, Victoria Udalova

**Affiliations:** 1 US Census Bureau; 2 Department of Epidemiology and Population Health, Stanford School of Medicine, Stanford University, Stanford, California; 3 Department of Medicine, Division of Primary Care and Population Health, Stanford School of Medicine, Stanford University, Stanford, California; 4 Department of Pediatrics, Stanford School of Medicine, Stanford, California; 5 Department of Health Policy, Stanford School of Medicine, Stanford University, Stanford, California; 6 Department of Sociology, Stanford University, Stanford, California; 7 American Board of Family Medicine, Lexington, Kentucky

## Abstract

**Importance:**

Area-based measures of social risk are increasingly being used in policy applications in the US. While several have been demonstrated to be predictive of health and mortality in the general population, there is a need to identify area-based measures that are most reliable for policy applications, including measures that are associated with health and mortality consistently across subpopulations.

**Objective:**

To compare the relative strength with which area and individual social risk measures are correlated with health outcomes and mortality, and the extent to which these associations are consistent across race, ethnicity, rurality, age, and gender.

**Design, Setting, and Participants:**

This cross-sectional study included a sample of patients from primary care clinics across all 50 states that are part of the PRIME registry using electronic health records (2019-2021) linked to US Census Bureau restricted-use data at the individual level from 947 US primary care practices.

**Exposures:**

Eight commonly used area based measures of social risk were examined: (1) Social Deprivation Index, (2) Social Vulnerability Index, (3) Area Deprivation Index (from University of Wisconsin), (4) Area Deprivation Index (constructed using Gophal Singh’s original design), (5) Neighborhood Stress Score, (6) Index of Concentration at the Extremes for race and income, (7) French Index of Social Deprivation, and (8) the Community Resilience Estimates. Individual socioeconomic measures of education, poverty, and occupation were also examined.

**Main Outcomes and Measures:**

Hypertension, diabetes, and chronic kidney disease derived from electronic health records, and mortality from the Census Numident.

**Results:**

Data from 2 801 000 patients were analyzed. Among these, 44% were male individuals and 56% were female individuals; 20% were younger than 25 years, 23% were aged 25 to 44 years, 30% were aged 45 to 64 years, and 27% were aged 65 years and older; 0.5% were American Indian or Alaskan Native, 2.1% Asian, 7.6% Black, 0.2% Native Hawaiian and Pacific Islander, 0.03% were 2 or more races, and 70% were White. Area-based measures of social risk were generally better predictors of hypertension, diabetes, and chronic kidney disease, whereas individual socioeconomic measures were generally better predictors of mortality. The strongest predictor across health outcomes was the Area Deprivation Index, and that Gopal Singh’s version was the most equitably predictive across rural areas and across all racial and ethnic subgroups.

**Conclusions and Relevance:**

In this cross-sectional study, area-based measures predicted health outcomes better than individual socioeconomic measures, and generally predicted health equitably across subpopulations; thus, their use should be considered in conjunction or instead of using individual-level measures for selected health policy applications.

## Key Points

**Question:**

How well do area-based measures of social risk, compared with individual level socioeconomic measures, predict health outcomes and mortality, overall, and by race, ethnicity, rurality, age, and gender?

**Findings:**

This cross-sectional study including more than 2.8 million patients evaluated 8 area-based measures of social risk for health and found that the Area Deprivation Index was the most consistent predictor of health outcomes and mortality across different subpopulations compared with other area- and individual-level social risk measures.

**Meaning:**

Area-based measures of social risk consistently capture meaningful aspects of health-related social risk across subpopulations, often outperforming individual socioeconomic measures, thus can be considered for broader policy applications, such as adjusting health care payments to meet social needs.

## Teaser

This cross-sectional study compares the relative strength with which area and individual social risk measures correlate with health outcomes and mortality, and the extent to which these associations are consistent across race, ethnicity, rurality, age, and gender.

## Introduction

Measurement of individual- and area-based social risks, which are associated with poor health outcomes, is critical for efficiently guiding services and resources for improving patient and population health. There is growing interest in using social risk information for policy applications, such as payment adjustment to clinicians and practices,^1^ equitably allocating vaccines,^2^ and allocation of government resources relating to health more generally.^3^ For these types of applications, a general measure is needed, one that captures the broad concept of social risk, and is also associated with a range of health outcomes, a task distinct from the development of clinical prediction models that are focused on specific health outcomes.^4^ As an example of the need for validated, broad social risk measures, the Centers for Medicare & Medicaid Services and the state of Maryland have incorporated an area-based measure of social risk into payment adjustment models, and 7 states are using a combination of individual socioeconomic measures from electronic health record (EHR) data and area-based measures derived from US Census Bureau data for payment adjustment.^5,6^

Social risk is distinct from other similar concepts (eg, social determinants of health, social needs, social deprivation) because it is meant to capture social exposures that are damaging to health in a comprehensive way.^7^ There are 2 broad types of social measures with which to capture social risk, individual and area based. However, there has not been a comprehensive, national, empirical analysis of the relative merits of how strongly each of these measures are related to health outcomes and mortality. This is important given the dozens of measures that are currently available.^8,9^ In addition, it is not clear whether these measures capture social needs better for some population subgroups. To address health and health care needs without contributing to inequities, it is critical that measures used for resource allocation work equally well in all subgroups of the population. While social policies in the US have typically been determined based on individual needs, frequently defined by income, there are several advantages to assessing needs based on area characteristics that have driven most agencies toward using this type of measure, rather than individual measures. Area-based measures may be easier and less costly to implement and may be less prone to errors and fraud. They are also closer to some of the theoretical underpinnings of social risk, which identify it as a multidimensional concept, rather than just income deprivation.^10^ Individual measures may also not be as relevant for certain portions of the population (eg, education for individuals younger than 25 years). Finally, they move the conceptual driver of social risk out of the individual to the context in which the individual resides. For these reasons, both the United Kingdom and New Zealand have successfully used area-based social risk measures to adjust health care resources, rather than relying on individual measures.^11^

There are, however, concerns about area-based measures. There is socioeconomic heterogeneity within the small areas typically used (census block group and census tract) for area-based socioeconomic measures. There are thus theoretical and empirical concerns that due to the mismatch between the actual social needs of a household compared with the average household needs in an area, the risk for patients may not be well addressed by area-based measures. Although these issues and the current extent of the use of area-based measures of social risk has been reviewed in a recent report,^5^ there was a notable lack of empirical evidence about the accuracy of area based or individual measures for predicting health outcomes. In addition, any assessment of the strength of prediction of health outcomes must assess how equitable these are across meaningful social domains (race and ethnicity, rurality, age, and gender).

The first goal of this analysis was thus to take the most common area-based measures of social risk, at the levels of geography most used for these measures, the census block group and census tract, and examine how well they predict common health outcomes and mortality compared with individual social measures. The second goal was to examine the relative strength of prediction across meaningful social characteristics.

## Methods

### Data

This research relied on data from the US Census Bureau for social measures and mortality data, and a national primary care registry for health outcomes. Area-based measures were calculated from publicly available American Community Survey (ACS) 5-year estimates (2016-2020). Individual socioeconomic measures were derived from restricted ACS microdata (2005-2022). Health outcomes were constructed from data derived from EHRs as part of the American Family Cohort (AFC) data, which includes patients with public and private insurance.^12^ Finally, mortality data were derived from date of death information in the Census Numident File. We used anonymized person-level identifiers (Protected Identification Keys [PIKs]) to link the EHR data with restricted individual-level data from the ACS (2005-2022).^13^ The study was approved by the Stanford University Panel on Medical Human Subjects (69891). Our reporting followed the Strengthening the Reporting of Observational Studies in Epidemiology (STROBE) reporting guidelines.

### Exposures

We examined 8 commonly used area-based measures of social risk using the publicly available 2016 to 2020 American Community Survey 5-year estimates: (1) Social Deprivation Index (SDI),^14^ (2) Social Vulnerability Index (SVI),^15^ (3) Area Deprivation Index (ADI-GS), as originally developed by Gophal Singh,^16^ (4) Area Deprivation Index (ADI-UW),^17^ as available from the University of Wisconsin, (5) Neighborhood Stress Score (NSS7),^18^ (6) Index of Concentration at the Extremes (ICE) for race and ethnicity and income^19^ with 1 measure comparing income between non-Hispanic White and Black populations (ICE wb-inc) and 1 measure examining incomes between non-Hispanic White and persons of color (ICE wpc-inc), (7) French Deprivation Index (FDep),^20^ and (8) Community Resilience Estimates (CRE).^21^ This list includes the 2 measures, the ADI and SDI, that were recommended based on a recent scoping review of the literature.^5^ The ADI-GS corrects for the lack of standardization that has been noted for the ADI-UW.^22,23^ The ICE measures are used to capture structural racism,^24^ and the FDep is used for measurement of social deprivation across a variety of European contexts, yet unlike the United Kingdom or New Zealand measures used for payment adjustment, it can be more easily adapted to the US context based on Census Bureau data. The CRE measures social vulnerability to disasters and provides estimates of the total number of people living in a community by the number of risk factors for all neighborhoods (tracts) using 1-year ACS and small area estimation techniques at the individual level. Except for ICE measures and CRE (for which block group is not available), all area based measures are examined at 2 levels of aggregation: census block group and census tract, for a total of 15 area-based measures. Consistent with the literature, we created quintiles for each measure and present our main models comparing prediction of health and mortality for the most deprived vs the least deprived quintiles. Each measure was coded so that higher levels indicate more socially deprived areas. eTable 1 in Supplement 1 shows the distribution of patient characteristics and health outcomes for AFC patients overall, AFC patients matched to ACS, the analytic sample, and the enhanced analytic sample using address identifiers (Master Address File Identifiers [MAFIDs]) obtained from the Master Address File—Auxiliary Reference File (MAF-ARF). Although sample size was smaller after matching, the distribution of characteristics is similar across samples (eTable 1 in Supplement 1).

In addition to area-based measures of social risk, we examined 3 individual level measures of social risk from the ACS: (1) educational attainment, (2) household poverty, and (3) occupational class. Educational attainment is coded for individuals 25 years or older at the time of response to ACS and includes less than high school, high school, some college, and at least a bachelor’s degree. Occupational class is coded for individuals 16 years or older who were reported being employed in the past 5 years and includes the following groups: white collar and professional, white collar and semiroute, blue collar and high skill, and blue collar that is semiroutine.^25^ Poverty index measures income as a function of poverty thresholds for related households, for all households where income and household size were obtained.

### Outcomes

The analyses focus on 3 health conditions and mortality. Health conditions were chosen to capture a range of important conditions encountered in primary care practices: (1) hypertension, (2) diabetes, and (3) chronic kidney disease. The 3 health outcomes were derived from longitudinal EHR data and were defined by having 1 or more diagnosis codes related to that condition in the 3-year period for which the AFC data are observed (2019-2021). Among patients who were coded as having 1 of these conditions, between 70% and 80% had 2 or more diagnosis codes for that condition. For patients with more than 1 health condition, their observation contributed to each health outcome independently. We also examined all-cause mortality, derived from the Census Numident file based on the Social Security Administration death dates.

### Statistical Models

Separate models were fit with outcomes noted as the dependent variables (4 models), in separate models with exposures (18 models) as predictors, for a total of 72 models. Odds ratios and 95% CIs were compared across key domains of predictors, with a focus of interpretation on whether particular area-based measures and individual measures are more predictive of outcomes. A second key focus for inference is comparing whether individual measures are more predictive than area-based measures. We also fit models controlling for age, gender, race and ethnicity, and rurality to examine whether the general pattern of association differs. All of this information was obtained from the EHRs. Race and ethnicity were most typically reported by participants, but EHR data in some cases may have been reported by the clinician. Race was coded to form a crude measure capturing broad differences in social exposures in the categories of American Indian and Alaska Native, Asian, Black, Native Hawaiian and Pacific Islander, White, 2 or more races, and missing. Ethnicity was categorized as Hispanic, non-Hispanic, and missing. Rurality was based on Rural-Urban Commuting Area (RUCA) codes as defined by the Federal Office of Rural Health Policy at the Health Resources & Services Administration.^26^ For each of the 72 models fit previously, we examined effect modification by age, gender, race, ethnicity, and rurality.

## Results

**Figure 1.**
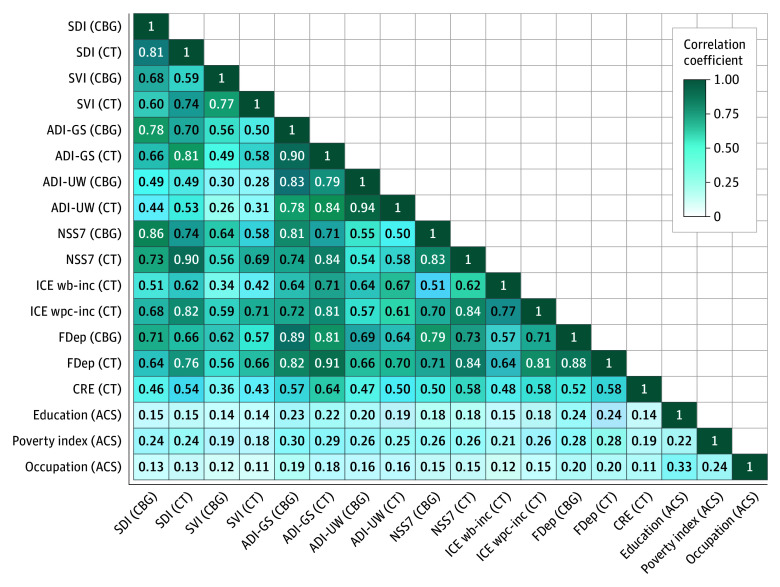
Correlations Between Area-Based Measures at the Census Block Group (CBG) and Census Tract (CT) and Individual Measures of Social Risk Pearson correlation coefficients (all correlations are *P* < .001). The Census Bureau has reviewed this data product to ensure appropriate access, use, and disclosure avoidance protection of the confidential source data used to produce this product (Data Management System number: P-7532672, disclosure review board approval number: CBDRB-FY24-POP001-0090). Source: American Family Cohort (AFC) Data 2019 to 2021; American Community Survey (2005-2022). ACS indicates the American Community Survey; ADI-GS indicates Area Deprivation Index–Gophal Singh; ADI-UW, Area Deprivation Index–University of Wisconsin; CBG, Census Block Group; CRE, Community Resilience Estimates; CT ,Census Tract; FDep, French deprivation index; ICE wb-inc, Index of concentration at the extremes–income between Non-Hispanic White and Black populations; ICE wpc-inc, Index of concentration at the extremes–incomes between non-Hispanic White and persons of color; NSS7, Neighborhood Stress Score; SDI, Social Deprivation Index; SVI, Social Vulnerability Index.

Figure 1 presents a correlation matrix demonstrating the extent to which different area-based and individual measures of social risk were correlated with each other (*P* < .001). Correlations among area-based measures ranged from 0.26 to 0.94. Area-based and individual measures were weakly correlated with each other, between 0.11 and 0.33, indicating that, although the directionality of such measures were consistent for capturing social needs, they were unique in the variance that each is capturing. This finding further motivated our comparison and careful consideration of which area-based measures were used, and the extent to which they captured similar health risks as individual measures.

**Figure 2.**
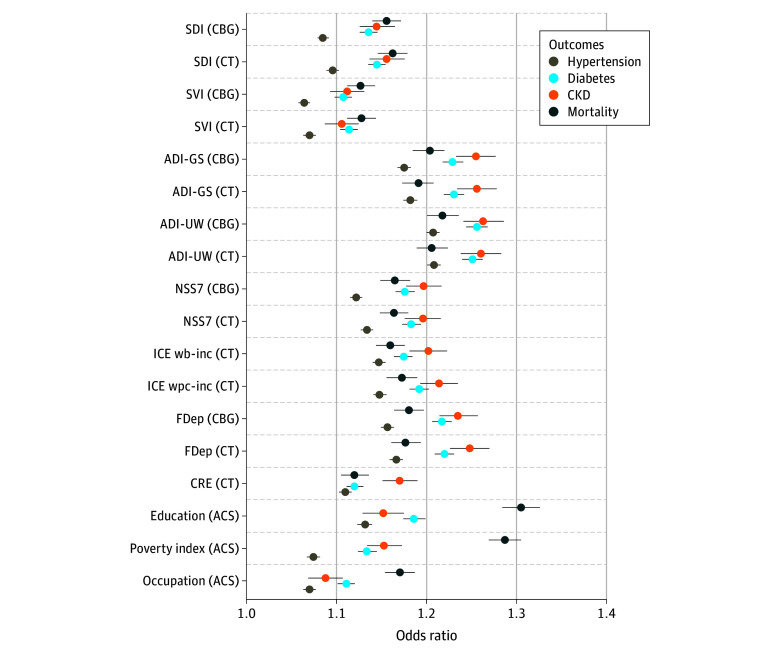
Odds Ratios for Associations Between Area-Based and Individual Measures of Social Risk With Health Outcomes and Mortality CKD indicates chronic kidney disease; CBG, census block group; CT, census tract. Models adjusted for age, race and ethnicity, gender, and rurality. The Census Bureau has reviewed this data product to ensure appropriate access, use, and disclosure avoidance protection of the confidential source data used to produce this product. (Data Management System number: P-7532672, disclosure review board approval number: CBDRB-FY24-POP001-0090). Source: American Family Cohort (AFC) data 2019 to 2021; American Community Survey (ACS) (2005-2022); census numident (Q3, 2023). ACS indicates the American Community Survey; ADI-GS indicates Area Deprivation Index–Gophal Singh; ADI-UW, Area Deprivation Index–University of Wisconsin; CBG, Census Block Group; CRE, Community Resilience Estimates; CT ,Census Tract; FDep, French deprivation index; ICE wb-inc, Index of concentration at the extremes–income between Non-Hispanic White and Black populations; ICE wpc-inc, Index of concentration at the extremes–incomes between non-Hispanic White and persons of color; NSS7, Neighborhood Stress Score; SDI, Social Deprivation Index; SVI, Social Vulnerability Index.

Figure 2 presents odds ratios (ORs) for associations of area-based measures of social risk (at the census block group [CBG] and census tract [CT] levels) and individual social risk measures with health and mortality, in models adjusting for age, race and ethnicity, gender, and rurality. eTable 2 in Supplement 1 presents the coefficients and 95% CIs for Figure 2, along with coefficients from unadjusted models. There were positive, consistent, meaningful, and statistically significant associations between level of social need captured by area-based measures with prevalence of hypertension, diabetes, chronic kidney disease, and incidence of mortality. Within this consistent prediction, however, there were differences in magnitude by area-based and individual measures, where 95% CIs were nonoverlapping. The strongest area-based predictors of health outcomes were both versions of the ADI, and additionally the FDep for predicting chronic kidney disease. Among individual measures, both education and poverty index were stronger predictors of outcomes than occupation. Although these 2 individual measures were stronger predictors of mortality than area-based measures, area-based measures were generally stronger predictors of the health conditions than individual measures. eTable 3 in Supplement 1 presents coefficients from area-based and individual measures by quintile of the distribution, demonstrating that these differences in prediction are not due to the standard categorization of area-based and individual measures. eFigure 1 and eTable 4 in Supplement 1 present coefficients from unadjusted models where all census tract measures are examined as deciles, demonstrating a fairly linear relation across levels.

**Figure 3.**
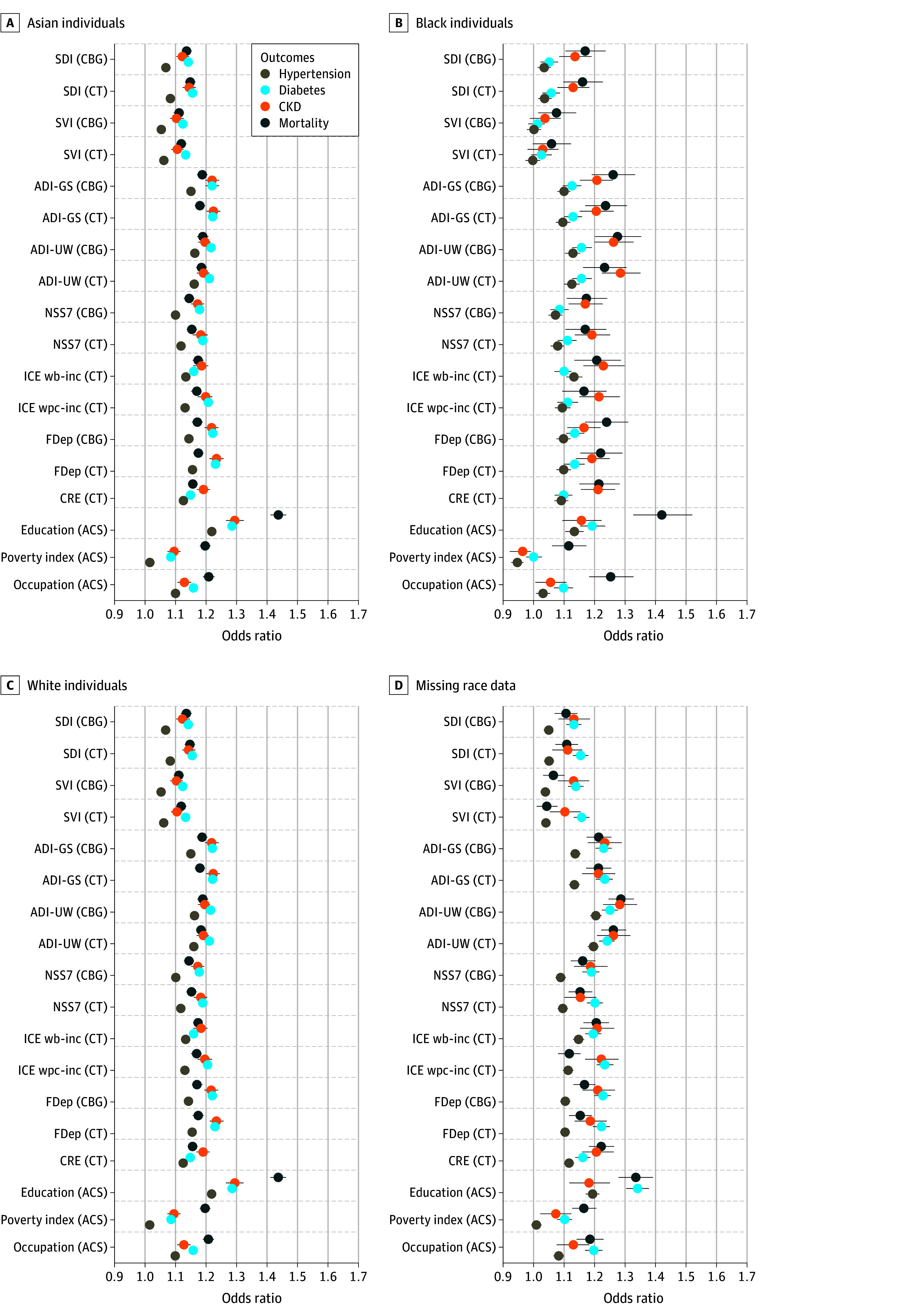
Odds Ratios for Associations Between Area and Individual Measures of Social Risk With Health Outcomes and Mortality, Stratified by Race CKD indicates chronic kidney disease; CBG,census block group; CT, census tract. Models are unadjusted. The Census Bureau has reviewed this data product to ensure appropriate access, use, and disclosure avoidance protection of the confidential source data used to produce this product (Data Management System number: P-7532672, disclosure review board approval number: CBDRB-FY24-POP001-0090). Source: American Family Cohort (AFC) data 2019 to 2021; American Community Survey (ACS) (2005-2022); Census Numident (Q3, 2023). ACS indicates the American Community Survey; ADI-GS indicates Area Deprivation Index–Gophal Singh; ADI-UW, Area Deprivation Index–University of Wisconsin; CBG, Census Block Group; CRE, Community Resilience Estimates; CT ,Census Tract; FDep, French deprivation index; ICE wb-inc, Index of concentration at the extremes–income between Non-Hispanic White and Black populations; ICE wpc-inc, Index of concentration at the extremes–incomes between non-Hispanic White and persons of color; NSS7, Neighborhood Stress Score; SDI, Social Deprivation Index; SVI, Social Vulnerability Index.

**Figure 4.**
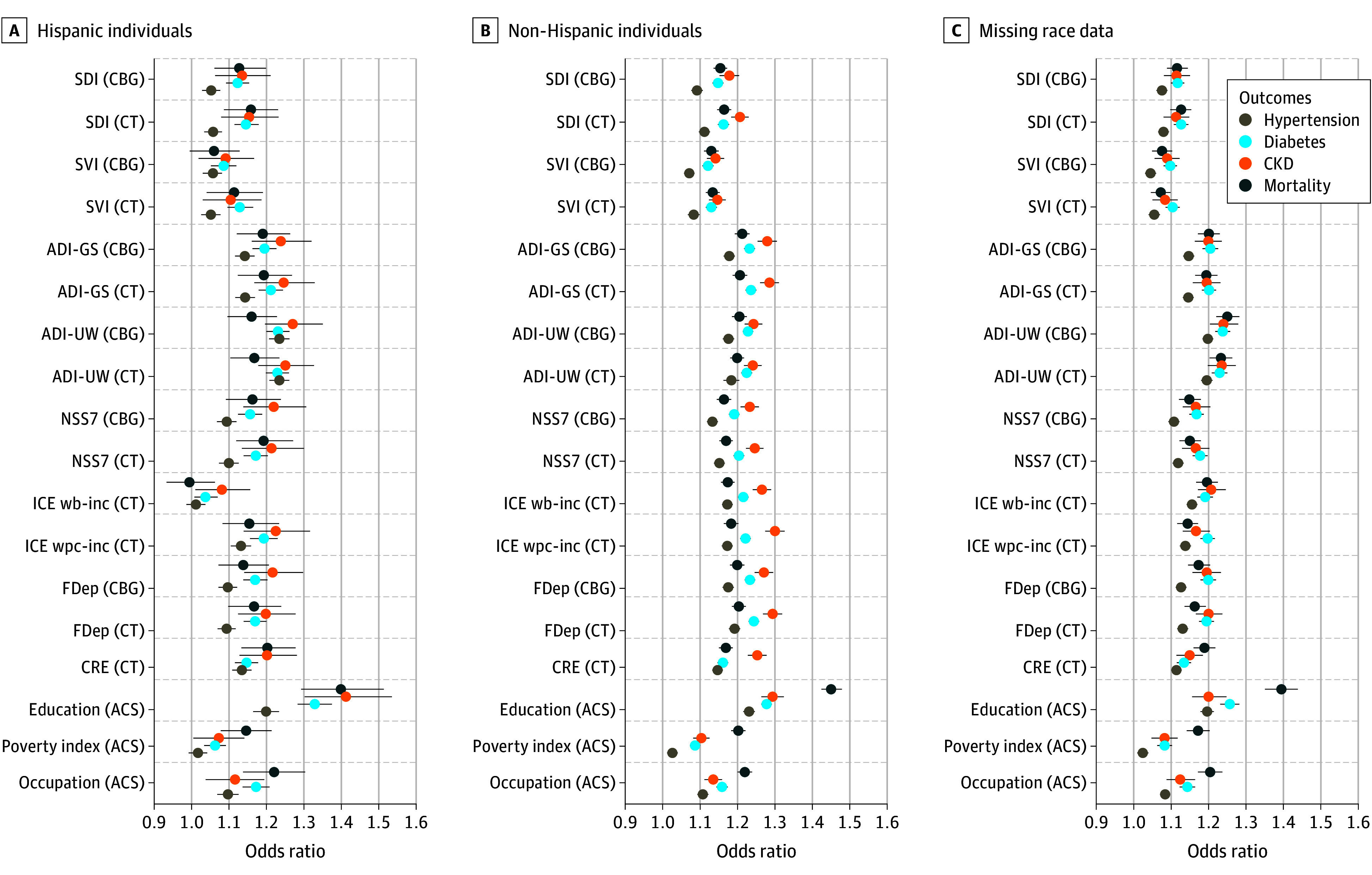
Odds Ratios for Associations Between Area and Individual Measures of Social Risk With Health Outcomes and Mortality, Stratified by Ethnicity CKD indicates chronic kidney disease; CBG, census block group; CT,census tract. Models are unadjusted. The Census Bureau has reviewed this data product to ensure appropriate access, use, and disclosure avoidance protection of the confidential source data used to produce this product (data management system number: P-7532672, disclosure review board approval number: CBDRB-FY24-POP001-0090). Source: American Family Cohort (AFC) data 2019 to 2021; American Community Survey (ACS) (2005-2022); Census Numident (Q3, 2023). ACS indicates the American Community Survey; ADI-GS indicates Area Deprivation Index–Gophal Singh; ADI-UW, Area Deprivation Index–University of Wisconsin; CBG, Census Block Group; CRE, Community Resilience Estimates; CT ,Census Tract; FDep, French deprivation index; ICE wb-inc, Index of concentration at the extremes–income between Non-Hispanic White and Black populations; ICE wpc-inc, Index of concentration at the extremes–incomes between non-Hispanic White and persons of color; NSS7, Neighborhood Stress Score; SDI, Social Deprivation Index; SVI, Social Vulnerability Index.

Figure 3 and Figure 4 present the results examining differences by race (Figure 3) and ethnicity (Figure 4). Across most racial and ethnic groups, area-based measures and individual socioeconomic measures consistently reported statistically significant and meaningful estimates of association similar to population averages, suggesting that area-based social measures can be used with equal validity across different racial and ethnic populations. The notable exception to this is the weaker prediction of health and mortality for the ADI-UW, ICE wb-inc, and the CRE in the Asian population, and the notably better prediction of these measures in the population missing information on race. eTable 5 (race) and eTable 6 (ethnicity) in Supplement 1 also present coefficients from unadjusted models.

**Figure 5.**
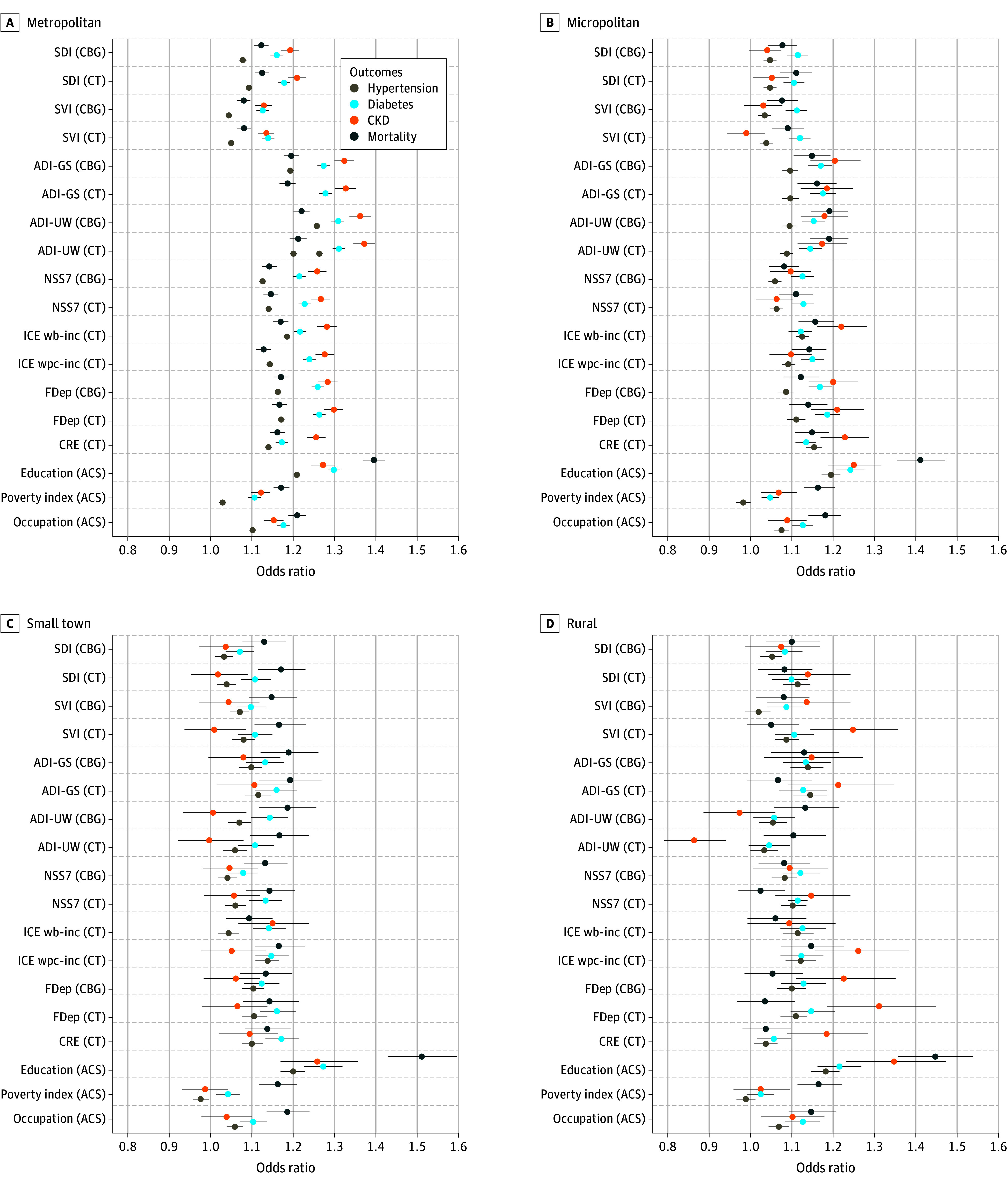
Odds Ratios for Associations Between Area and Individual Measures of Social Risk With Health Outcomes and Mortality, Stratified by Rurality CKD indicates chronic kidney disease; CBG, census block group; CT, census tract. Models are unadjusted. The Census Bureau has reviewed this data product to ensure appropriate access, use, and disclosure avoidance protection of the confidential source data used to produce this product (Data Management System number: P-7532672, disclosure review board approval number: CBDRB-FY24-POP001-0090). Source: American Family Cohort (AFC) data 2019 to 2021; American Community Survey (ACS) (2005-2022); Census Numident (Q3, 2023). ACS indicates the American Community Survey; ADI-GS indicates Area Deprivation Index–Gophal Singh; ADI-UW, Area Deprivation Index–University of Wisconsin; CBG, Census Block Group; CRE, Community Resilience Estimates; CT ,Census Tract; FDep, French deprivation index; ICE wb-inc, Index of concentration at the extremes–income between Non-Hispanic White and Black populations; ICE wpc-inc, Index of concentration at the extremes–incomes between non-Hispanic White and persons of color; NSS7, Neighborhood Stress Score; SDI, Social Deprivation Index; SVI, Social Vulnerability Index.

Figure 5 presents the results from models examining differences in prediction by rurality. In general, both area-based and individual measures were worse at predicting health outcomes and mortality in smaller towns, rural areas, and micropolitan areas compared with metropolitan areas. The most dramatic difference for this was for the ADI-UW measure, which did not predict outcomes in small town and rural areas for more common outcomes like hypertension. For chronic kidney disease, the ADI-UW measure had a coefficient showing a protective effect, but with 95% CIs that included the null of no association. However, across all 3 health outcomes, the ADI-GS was the best predictor, with overlapping 95% CIs with individual education for some outcomes, or close to as predictive as individual-level education for other outcomes. For mortality, both ADI measures were similarly predictive. eTable 7 in Supplement 1 presents coefficients from Figure 5.

We also examined differences in prediction stratified by gender and age, presented in eTables 8 and 9 in Supplement 1, respectively. Across gender, prediction was generally better for women, but in most cases these differences were small. For age, similar associations existed across age groups, with the expected attenuation of associations at 65 years and older, for both area-based and individual measures, as is observed in most of the epidemiologic literature.^27^

## Discussion

One important aspect of validating area-based measures of social risk for program administration and policy applications is examining the extent to which these measures predict health outcomes overall, and the extent to which this prediction is consistent across socially meaningful subgroups of the population. Critical to this endeavor is the examination of prediction of a range of health outcomes, including mortality because these social risk measures are intended to be used for capturing a higher risk of disease overall, rather than for prediction of specific outcomes. It is also important to demonstrate that, given the logistical and theory-based advantages of using area-based measures, that they predict health with a degree of magnitude similar to individual measurement of social risk, which often can be difficult or costly to obtain. We were able to answer these questions with a novel linkage between individual-level Census Bureau and EHR data.^13^ Our analyses demonstrate the validity of using area-based measures for the purposes of identifying at-risk communities and supporting health programs aimed at promoting equitable care.

These findings demonstrate that area measures are consistently associated with health and mortality across diverse population groups, and a range of area measures are useful and equitable for capturing social risk. Although prior work has compared 2 measures (ADI and SVI) with each other,^9^ our findings compared multiple measures, used a considerably larger sample size, included a population with both private and public insurance, and also compared measures at 2 levels of aggregation (census tract and block group). We found that among area-based measures, the ADI-GS was most equally predictive across groups. The notable differences between the ADI-GS and the ADI-UW are structural—the ADI-GS standardizes all variables so that they are on the same scale, preventing inputs measured over a larger range from having a greater influence than their weights; and in effect the ADI-GS is more equitably predictive for estimating deprivation in small towns and rural areas. We did not distinguish markedly greater prediction by level of census geography, with prediction similar between the smaller less heterogenous census block group compared with the larger census tract. Our data thus suggest the use of the ADI-GS at either measure of geography is appropriate, which is similar to prior findings specific to the state of Massachusetts using data from 1990.^28^ This is notably different from the ADI-UW, which although highly predictive in several groups, is sensitive to rurality, consistent with prior work on the ADI-UW.^29^ This consideration is important, because for policy purposes it is critical that measures used would be reliable for allocating resources across all areas where the measure would be used. Area based measures must offer reliable prediction in different geographic areas, which is not the case with the ADI-UW.

Association, however, is just 1 element of the validity of measures of social risk. Another key aspect is the content validity—that they do indeed capture socially relevant aspects of affluence and deprivation. We only included measures that were intended to capture social risk. We did not include area-based measures that were developed purely for prediction of health outcomes or mortality.^30^ Some of these measures, for example, included factors like the age composition of the population and close proxies for age, such as proportion of the population in long-term care facilities, which are highly predictive of health and mortality but are not measures of social risk.^5^ Another key consideration for interpreting our findings is for what purpose they will be applied. Area-based measures of social risk have a range of uses and decisions about the best measure may depend more heavily on the included constructs rather than predictive validity. Finally, the use of measures should involve collaboration with community and other stakeholders. Although we have had prior engagement with such stakeholders as part of this work, we view these analyses as a continuing iterative process, and that this work can help contribute to such ongoing discussions about the appropriate use of area-based measures of social risk for particular use cases.

### Limitations

This cross-sectional study had several limitations. Although the underlying data from the PRIME registry is arguably the most representative EHR data that currently exists in the US, it is not based on a probability sample. In addition, health outcomes, as recorded in the EHR, may be recorded with error. Despite these limitations, we view these data as an excellent choice for this initial comprehensive validation work because many use cases for area-based measures of social risk will rely on population-based EHR data.

## Conclusions

Our intent is that the validation approach that we took here should be replicated with other sources of data subject to different types of selection mechanisms to continue the process of validation. Not only will a continuous evaluation approach aid in the triangulation of true associations in the population, but will also allow for researchers to track how these associations may change over time, which is expected given the contextually contingent nature of social risk.^31^

## Notes

Supplement 1. eMethods eTable 1. Characteristics of patients in AFC data eTable 2. Coefficients Plotted in Figure 2 (Adjusted Models), Compared to Coefficients from the Same Models but Without Covariate Adjustment (Unadjusted Models) eTable 3. Similar Coefficients Plotted in Figure 2 (Adjusted Models) but Social Measures are Categorical (Quintiles) eTable 4. Odds Ratios and 95% Confidence Intervals for Associations Between Area Measures (Deciles) of Social Risk with Health Outcomes and Mortality eTable 5. Coefficients Plotted in Figure 3 (Race), Including Smaller Racial Groups (AIAN, NHPI, Two or More Races) eTable 6. Coefficients Plotted in Figure 4 (Ethnicity) eTable 7. Coefficients Plotted in Figure 5 (Rurality) eTable 8. Odds Ratios and 95% Confidence Intervals for Associations Between Area and Individual Measures of Social Risk with Health Outcomes and Mortality, Stratified by Gender eTable 9. Odds Ratios and 95% Confidence Intervals for Associations Between Area and Individual Measures of Social Risk with Health Outcomes and Mortality, Stratified by Age eFigure 1. Odds Ratios and 95% Confidence Intervals for Associations Between Area Measures (Deciles) of Social Risk with Health Outcomes and Mortality eFigure. Odds Ratios and 95% Confidence Intervals for Associations Between Area Measures (Deciles) of Social Risk with Health Outcomes and Mortality

Supplement 2. Data Sharing Statement
