# Supplementary material for: Validating 8 Area-Based Measures of Social Risk for Predicting Health and Mortality
Source: JAMA Health Forum. 2025 Aug 15;6(8):e252669. doi: 10.1001/jamahealthforum.2025.2669 (PMC12357199; doi:10.1001/jamahealthforum.2025.2669)
Supplement: Supplement 1. — eMethods eTable 1. Characteristics of patients in AFC data eTable 2. Coefficients Plotted in Figure 2 (Adjusted Models), Compared to Coefficients from the Same Models but Without Covariate Adjustment (Unadjusted Models) eTable 3. Similar Coefficients Plotted in Figure 2 (Adjusted Models) but Social Measures are Categorical (Quintiles) eTable 4. Odds Ratios and 95% Confidence Intervals for Associations Between Area Measures (Deciles) of Social Risk with Health Outcomes and Mortality eTable 5. Coefficients Plotted in Figure 3 (Race), Including Smaller Racial Groups (AIAN, NHPI, Two or More Races) eTable 6. Coefficients Plotted in Figure 4 (Ethnicity) eTable 7. Coefficients Plotted in Figure 5 (Rurality) eTable 8. Odds Ratios and 95% Confidence Intervals for Associations Between Area and Individual Measures of Social Risk with Health Outcomes and Mortality, Stratified by Gender eTable 9. Odds Ratios and 95% Confidence Intervals for Associations Between Area and Individual Measures of Social Risk with Health Outcomes and Mortality, Stratified by Age eFigure 1. Odds Ratios and 95% Confidence Intervals for Associations Between Area Measures (Deciles) of Social Risk with Health Outcomes and Mortality eFigure. Odds Ratios and 95% Confidence Intervals for Associations Between Area Measures (Deciles) of Social Risk with Health Outcomes and Mortality [file jamahealthforum-e252669-s001.pdf]

Supplemental Online Content

Limburg A, Rehkopf DH, Gladish N, Phillips RL, Udalova V. Validating 8 area-based measures of social risk for predicting health and mortality. *JAMA Health Forum*. 2025;6(8):e252669. doi:10.1001/jamahealthforum.2025.2669

eMethods
eTable 1. Characteristics of patients in AFC data
eTable 2. Coefficients Plotted in Figure 2 (Adjusted Models), Compared to Coefficients from the Same Models but Without Covariate Adjustment (Unadjusted Models)
eTable 3. Similar Coefficients Plotted in Figure 2 (Adjusted Models) but Social Measures are Categorical (Quintiles)
eTable 4. Odds Ratios and 95% Confidence Intervals for Associations Between Area Measures (Deciles) of Social Risk with Health Outcomes and Mortality
eTable 5. Coefficients Plotted in Figure 3 (Race), Including Smaller Racial Groups (AIAN, NHPI, Two or More Races)
eTable 6. Coefficients Plotted in Figure 4 (Ethnicity)
eTable 7. Coefficients Plotted in Figure 5 (Rurality)
eTable 8. Odds Ratios and 95% Confidence Intervals for Associations Between Area and Individual Measures of Social Risk with Health Outcomes and Mortality, Stratified by Gender
eTable 9. Odds Ratios and 95% Confidence Intervals for Associations Between Area and Individual Measures of Social Risk with Health Outcomes and Mortality, Stratified by Age
eFigure 1. Odds Ratios and 95% Confidence Intervals for Associations Between Area Measures (Deciles) of Social Risk with Health Outcomes and Mortality
eFigure. Odds Ratios and 95% Confidence Intervals for Associations Between Area Measures (Deciles) of Social Risk with Health Outcomes and Mortality

This supplemental material has been provided by the authors to give readers additional information about their work.

© 2025, Limburg A, et al. *JAMA Health Forum*.

Acronym List

ACS	American Community Survey
ADI-GS	Area Deprivation Index - Gophal Singh
ADI-UW	Area Deprivation Index - University of Wisconsin
AFC	American Family Cohort
CBG	Census block group
CRE	Community Resilience Estimates
CT	Census tract
EHR	Electronic health records
FDep	French Deprivation Index
ICE wb-inc	Index of Concentration at the Extremes, comparing income between Non-Hispanic White and Black populations
ICE wpc-inc	Index of Concentration at the Extremes, comparing income between Non-Hispanic White and persons of color
MAF-ARF	Master Address File-Auxiliary Reference File
MAFID	Master Address File identifier
NSS7	Neighborhood Stress Score
PIK	Protected Identification Key
PVS	Person Identification Validation System
RUCA	Rural-Urban Community Area
SDI	Social Deprivation Index
SSN	Social Security number
SVI	Social Vulnerability Index

Data Providence
This research is based on a collaboration between the American Board of Family Medicine (ABFM), Stanford University, and the Enhancing Health Data (EHealth) Program at the Census Bureau. The purpose of this collaboration is to conduct innovative linkages between EHRs and restricted Census Bureau microdata as a means of producing high quality statistics and conducting health research that marries clinical and social indicators in novel ways. Secure disclosure of EHRs to the Census Bureau satisfies the data owner's obligation to protect these data as required by the Health Insurance Portability and Accountability Act (HIPAA). Title 13 of the U.S. Code authorizes the Census Bureau to collect information from other entities and requires the Census Bureau to keep the information confidential and use it only for statistical purposes. Any aggregate statistics produced using EHRs and Census Bureau microdata is in full compliance with the robust disclosure avoidance techniques and reviewed and approved for public release by the Census Bureau's Disclosure Review Board. Creation of blended EHRs and Census Bureau data expands the type and quality of data products and research that can be created. This allows the public and policymakers to improve decision making by implementing policies that directly impact individuals' access to care, quality of care, and health outcomes.

Supplemental Methods for Calculating Social Risk Measures
Six measures of social risk were recreated using the 2016-2020 American Community Survey (ACS) 5-year estimates. The SDI, SVI, ADI, NSS7, and the FDep were made at the county, census tract (CT), and census block group (CBG) level. ICE was only created at the county and CT level as there were not appropriate tables available at the CBG level. Census tables were selected which best reflected the original variables used in the measures. Missing values were dealt with through imputing the median values. All analyses were done in the R environment (version 4.2.1) using RStudio (version 2023.06.0+421). The data was pulled into the R environment (version 4.2.1) using the get_acs function in the tidycensus package (version 1.2.2).

Social Vulnerability Index (SVI)
Calculation of the SVI has been documented in detail elsewhere: https://stacks.cdc.gov/view/cdc/134506. In summary, this measure was calculated by obtaining the percentage of individuals in each geographical

area for each individual variable. These percentage measures were then added together, and percentile ranked according to four themes: socioeconomic status, household composition and disability, minority status and language, and housing type and transportation. Finally, these four themes were then added together, and percentile ranked to produce the final SVI measure. The SVI produces a range from 0 to 1 where the closer to 1 the more vulnerable individuals are within the area.

Social Deprivation Index (SDI)
Production of the SDI is described in detail elsewhere: https://pubmed.ncbi.nlm.nih.gov/22816561/. To summarize, the variables composing the SDI were originally selected using factor analysis and a loading score threshold of > 0.6. Factor analysis was re-run with the subset of selected variables and the factor loadings were then used as weights to obtain scores for each geographical area. In the current analysis, variable selection was skipped as the same variables previously reported were used in the factor analysis to obtain the updated scores using the 2020 5-year AFS data. The factor analysis scores were scaled to ensure the SDI obtained a range of 0-100 where the closer an area to 100, the more socially deprived.

Area Deprivation Index (ADI) – University of Wisconsin-Madison
The ADI was originally made in 2003 (https://pmc.ncbi.nlm.nih.gov/articles/PMC1447923/) and then updated using the 2014-2018 ACS 5-year estimates to be calculated at the CBG level (https://pubmed.ncbi.nlm.nih.gov/25437404/; https://pubmed.ncbi.nlm.nih.gov/29949490/). As we wanted to investigate this measure at the CT and potentially county level, we reverse engineered the production of the ADI and compared it to the data available on their site. Version 2020 of the Neighborhood Atlas calculated ADI was accessed on 09 June 2023 from the Neighborhood Atlas website (https://www.neighborhoodatlas.medicine.wisc.edu/). The file entitled "US_2020_ADI_Census Block Group_v3.2.csv" was brought into the R environment using RStudio. To summarize how the ADI was produced, 17 census variables were used in a factor analysis where the variables were weighted using factor score coefficients obtained from Singh et al. 2003. As Kind et al. 2014 utilized the 2003 coefficients instead of reproducing them with the newer data, these coefficients were also utilized to calculate the ADI in the current study. These coefficients are multiplied against their raw, corresponding variables which are then added together. Subsequently, the ADI mean was arbitrarily set to 100 and standard deviation to 20 producing values where the higher the score, the higher the levels of deprivation. Comparison between the reverse engineered and University of Wisconsin ADI had a Pearson's correlation of 0.992, p-value ≤ 2.2x10-16, ICC = 0.992 (95% CI 0.991<ICC<0.992), and a root mean squared error = 3.71.

Area Deprivation Index – Gophal Singh Version
We created the veritable ADI based on the original ADI using 2016-2020 ACS 5-year estimates at the census tract level: https://pmc.ncbi.nlm.nih.gov/articles/PMC1447923/. This involves adjusting three of the 17 measures to reflect 2020 appropriate thresholds as Singh et al. did when calculating the 1990 versus the 1970 indexes: https://pmc.ncbi.nlm.nih.gov/articles/PMC1447923/. The three specific variable threshold changes are:

1. "Population aged ≥ 25 y with < 9 y of education" to "Population aged ≥ 25 y with at least high school diploma".
2. "Population aged ≥ 25 y with at least high school diploma" to "Population aged ≥ 25 y with at least a bachelor's degree".
3. Income disparity defined as "the log of 100 x ratio of number of households with < $10,000 income to number of households with ≥$50,000 income" to "the log of 100 x ratio of number of households with < $20,000 income to number of households with ≥$100,000 income".

The 17 variables are then imputed using the nearest neighbor imputation method: https://pmc.ncbi.nlm.nih.gov/articles/PMC3130338/. Neighbors are assigned based on contiguity type Queen where the median value of bordering census tracts, defined as spatial units which share a common edge or vertex, are used to impute the missing census tract value. Factor analysis was performed on population weighted, 2016-2020 ACS 5-year estimates to produce new variable weights for 2020 ADI creation, in line to Singh's methods in the original manuscript. This is contrary to what was done in the Neighborhood Atlas version which used weights produced from 1990 data. The resultant factor

analysis weights were then applied to population weighted and standardized data, summed, and scaled to range from 0-100 to produce the ADI.

Neighborhood Stress Score (NSS7)

The NSS7 was produced using seven census variables identified using principal components analysis (PCA): https://pubmed.ncbi.nlm.nih.gov/28783811/. Updated calculations simply use the measures previously identified without re-running a PCA. Currently, all variables were calculated into a percentage and then standardized using the formula z1 = (v1 – mean(v1))/SD(v1) where v = variable. All standardized variables are then summed together (S) and NSS7 = (S – mean(S))/SD(S). NSS7 used as a coefficient in a regression model should be interpreted as the increment to expected cost related to 1 SD increase in NSS7; therefore, the larger the value, the more vulnerable.

Index of Concentration at the Extremes (ICE)

ICE measures were calculated using this general formula as previously reported: (https://pmc.ncbi.nlm.nih.gov/articles/PMC4815605/):

$$ICE_i = (A_i - P_i)/T_i$$

Where Ai is the number of affluent individuals, Pi the number of deprived individuals, and Ti the total number of individuals in the population. So long as a given metric can be made dichotomous it can be included in this measure. Initially, we calculated the same ICE variables as Krieger et al., 2016, accounting for the proportion of Black versus Non-Hispanic White (ICE BW), the number of people whose income is ≤ 20th percentile of the US-wide median income (measured using the 2010, 2015, and 2020 ACS) versus those ≥ 80th percentile (ICE INC), and the number of Black individuals whose income is ≤ 20th percentile versus Non-Hispanic Whites whose income is ≥ 80th percentile (ICE wb-inc). To be inclusive of all minorities, two additional measures were made here; the number of Non-Hispanic White individuals versus minorities (ICE WNH), and the number of minorities whose income is ≤ 20th percentile versus Non-Hispanic White (persons of color) individuals whose income is ≥ 80th percentile (ICE wpc-inc).

French Deprivation Score (FDep)

The FDep was created by taking four census variables and performing PCA, taking the PC1 scores, and assigning them to the geographical areas. Rey et al. states that when run on French census data, PC1 explained approximately 68% of the variation: https://bmcpublichealth.biomedcentral.com/articles/10.1186/1471-2458-9-33.

eTable 1. Characteristics of patients in AFC data

	AFC Overall		AFC patients matched to ACS		Analytic sample		Enhanced analytic sample - with MAFIDs	
	N	%	N	%	N	%	N	%
Total	2,801,000	100.0	619,000	22.1	312,000	0.5	357,000	57.7
Gender								
Male	1,242,000	44.3	271,000	43.8	146,000	46.8	167,000	46.8
Female	1,557,000	55.6	348,000	56.2	166,000	53.2	189,000	52.9
Missing	2,200	0.1	400	0.1	200	0.1	200	0.1
Age (at last visit)								
<25	558,000	19.9	92,000	14.9	1,000	0.3	1,200	0.3
25-44	637,000	22.7	128,000	20.7	69,500	22.3	79,500	22.3
45-64	839,000	30.0	192,000	31.0	147,000	47.1	168,000	47.1
>=65	767,000	27.4	207,000	33.4	94,500	30.3	108,000	30.3
Race								
White	1,954,000	69.8	464,000	75.0	236,000	75.6	270,000	75.6
Black	213,000	7.6	35,000	5.7	17,000	5.4	19,500	5.5
AIAN	13,500	0.5	3,100	0.5	1,100	0.4	1,400	0.4
Asian	59,500	2.1	10,500	1.7	5,900	1.9	6,600	1.8
NHPI	5,100	0.2	1,000	0.2	550	0.2	600	0.2
Two or more races	850	0.0	150	0.0	80	0.0	90	0.0
Missing	556,000	19.9	105,000	17.0	51,000	16.3	58,000	16.2
Ethnicity								
Not Hispanic or Latino	1,730,000	61.8	406,000	65.6	208,000	66.7	237,000	66.4
Hispanic or Latino	277,000	9.9	45,000	7.3	21,000	6.7	24,000	6.7
Missing	794,000	28.3	168,000	27.1	83,000	26.6	96,000	26.9
Rurality (RUCA, zip code)								
Metropolitan	1,982,000	70.8	412,000	66.6	219,000	70.2	247,000	69.2
Micropolitan	417,000	14.9	97,500	15.8	46,500	14.9	53,000	14.8
Small town	257,000	9.2	63,500	10.3	28,000	9.0	33,000	9.2
Rural	136,000	4.9	45,000	7.3	19,000	6.1	23,000	6.4
Missing	9,100	0.3	1,800	0.3	<15	-	900	0.3
Health conditions								
Hypertension	620,000	22.1	154,000	24.9	84,000	26.9	97,500	27.3
Diabetes	258,000	9.2	63,000	10.2	33,500	10.7	38,500	10.8
Chronic kidney disease	77,000	2.7	20,000	3.2	8,300	2.7	9,400	2.6
All-cause mortality	161,000	5.7	40,500	6.5	13,000	4.2	15,000	4.2
Missing block group SDVI	379,000	13.5	85,000	13.7	-	-	-	-
Missing tract SDVI measures	378,000	13.5	85,000	13.7	-	-	-	-
Educational attainment (ACS)								

College +	-	140,000	22.6	101,000	32.4	114,000	31.9
Some College	-	130,000	21.0	86,000	27.6	98,500	27.6
High School	-	117,000	18.9	65,500	21.0	75,500	21.1
< High School	-	99,000	16.0	60,000	19.2	68,000	19.0
Missing	-	133,000	21.5	-	-	-	-
Poverty index (ACS)							
>=400%	-	273,000	44.1	169,000	54.2	192,000	53.8
300-399%	-	91,000	14.7	47,500	15.2	54,500	15.3
200-299%	-	98,500	15.9	46,000	14.7	52,500	14.7
100-199%	-	91,500	14.8	33,500	10.7	39,000	10.9
<100%	-	57,000	9.2	16,000	5.1	18,500	5.2
Missing	-	8,700	1.4	-	-	-	-
Occupation (ACS)							
White collar, professional	-	140,000	22.6	117,000	37.5	133,000	37.3
White collar, semi-routine	-	87,000	14.1	68,500	22.0	78,000	21.8
Blue collar, high school	-	30,500	4.9	23,500	7.5	27,500	7.7
Blue collar, semi-routine	-	144,000	23.3	103,000	33.0	118,000	33.1
Missing	-	217,000	35.1	-	-	-	-

Source: American Family Cohort (AFC) data 2019-2021; American Community Survey (2005-2022); Master Address File-Auxiliary Reference File (2018-2022); Census Numident (Q3, 2023).

Notes: AIAN = American Indian or Alaska Native; NHPI = Native Hawaiian or Pacific Islander; RUCA = Rural-Urban Commuting Area; ACS = American Community Survey. The Census Bureau has reviewed this data product to ensure appropriate access, use, and disclosure avoidance protection of the confidential source data used to produce this product (Data Management System (DMS) number: P-7532672, Disclosure Review Board (DRB) approval number: CBDRB-FY24-POP001-0090). Unweighted counts were rounded according to Census Bureau disclosure protocols to preserve data privacy and therefore may not sum perfectly due to rounding error.

eTable 2. Coefficients Plotted in Figure 2 (Adjusted Models), Compared to Coefficients from the Same Models but Without Covariate Adjustment (Unadjusted Models)

	Adjusted Models in Figure 2 (N=312,000)							
	Hypertension		Diabetes		Chronic Kidney Disease		Mortality	
	OR	95% CI	OR	95% CI	OR	95% CI	OR	95% CI
SDI (CBG)	1.09	(1.08, 1.09)	1.14	(1.13, 1.15)	1.15	(1.13, 1.17)	1.16	(1.14, 1.17)
SDI (CT)	1.1	(1.09, 1.1)	1.15	(1.14, 1.16)	1.16	(1.14, 1.18)	1.16	(1.15, 1.18)
SVI (CBG)	1.06	(1.06, 1.07)	1.11	(1.1, 1.12)	1.11	(1.09, 1.13)	1.13	(1.11, 1.14)
SVI (CT)	1.07	(1.06, 1.08)	1.11	(1.1, 1.12)	1.11	(1.09, 1.13)	1.13	(1.11, 1.14)
ADI-GS (CBG)	1.18	(1.17, 1.18)	1.23	(1.22, 1.24)	1.26	(1.23, 1.28)	1.2	(1.19, 1.22)
ADI-GS (CT)	1.18	(1.17, 1.19)	1.23	(1.22, 1.24)	1.26	(1.23, 1.28)	1.19	(1.17, 1.21)
ADI-UW (CBG)	1.21	(1.2, 1.22)	1.26	(1.24, 1.27)	1.26	(1.24, 1.29)	1.22	(1.2, 1.24)
ADI-UW (CT)	1.21	(1.2, 1.22)	1.25	(1.24, 1.26)	1.26	(1.24, 1.28)	1.21	(1.19, 1.22)
NSS7 (CBG)	1.12	(1.12, 1.13)	1.18	(1.17, 1.19)	1.2	(1.18, 1.22)	1.17	(1.15, 1.18)
NSS7 (CT)	1.13	(1.13, 1.14)	1.18	(1.17, 1.19)	1.2	(1.18, 1.22)	1.16	(1.15, 1.18)
ICE wb-inc (CT)	1.15	(1.14, 1.16)	1.18	(1.16, 1.19)	1.2	(1.18, 1.22)	1.16	(1.14, 1.18)
ICE wpc-inc (CT)	1.15	(1.14, 1.16)	1.19	(1.18, 1.2)	1.21	(1.19, 1.24)	1.17	(1.16, 1.19)
FDep (CBG)	1.16	(1.15, 1.16)	1.22	(1.21, 1.23)	1.24	(1.21, 1.26)	1.18	(1.16, 1.2)
FDep (CT)	1.17	(1.16, 1.17)	1.22	(1.21, 1.23)	1.25	(1.23, 1.27)	1.18	(1.16, 1.19)
CRE (CT)	1.11	(1.1, 1.12)	1.12	(1.11, 1.13)	1.17	(1.15, 1.19)	1.12	(1.11, 1.14)
Education (ACS)	1.13	(1.12, 1.14)	1.19	(1.17, 1.2)	1.15	(1.13, 1.18)	1.31	(1.28, 1.33)
Poverty index (ACS)	1.08	(1.07, 1.08)	1.13	(1.12, 1.15)	1.15	(1.13, 1.17)	1.29	(1.27, 1.31)
Occupation (ACS)	1.07	(1.06, 1.08)	1.11	(1.1, 1.12)	1.09	(1.07, 1.11)	1.17	(1.15, 1.19)
	**Unadjusted Models (N=312,000)**							
	Hypertension		Diabetes		Chronic Kidney Disease		Mortality	
	OR	95% CI	OR	95% CI	OR	95% CI	OR	95% CI
SDI (CBG)	1.07	(1.07, 1.08)	1.15	(1.14, 1.16)	1.15	(1.13, 1.17)	1.13	(1.11, 1.14)
SDI (CT)	1.09	(1.08, 1.09)	1.16	(1.15, 1.17)	1.17	(1.15, 1.19)	1.14	(1.12, 1.15)
SVI (CBG)	1.05	(1.04, 1.05)	1.12	(1.11, 1.13)	1.11	(1.1, 1.13)	1.09	(1.08, 1.1)
SVI (CT)	1.06	(1.05, 1.06)	1.13	(1.13, 1.14)	1.11	(1.1, 1.13)	1.09	(1.08, 1.1)
ADI-GS (CBG)	1.17	(1.16, 1.17)	1.23	(1.22, 1.24)	1.25	(1.23, 1.27)	1.2	(1.19, 1.22)
ADI-GS (CT)	1.17	(1.16, 1.18)	1.23	(1.22, 1.24)	1.25	(1.23, 1.27)	1.19	(1.18, 1.21)
ADI-UW (CBG)	1.19	(1.19, 1.2)	1.23	(1.22, 1.24)	1.25	(1.23, 1.27)	1.22	(1.2, 1.23)
ADI-UW (CT)	1.19	(1.19, 1.2)	1.23	(1.22, 1.24)	1.24	(1.22, 1.26)	1.21	(1.19, 1.22)
NSS7 (CBG)	1.11	(1.11, 1.12)	1.19	(1.18, 1.2)	1.21	(1.19, 1.23)	1.15	(1.13, 1.16)
NSS7 (CT)	1.13	(1.12, 1.13)	1.2	(1.19, 1.21)	1.21	(1.19, 1.23)	1.15	(1.14, 1.16)
ICE wb-inc (CT)	1.17	(1.16, 1.17)	1.19	(1.18, 1.2)	1.24	(1.22, 1.26)	1.18	(1.16, 1.19)
ICE wpc-inc (CT)	1.14	(1.13, 1.15)	1.22	(1.21, 1.23)	1.24	(1.22, 1.26)	1.15	(1.13, 1.16)
FDep (CBG)	1.15	(1.14, 1.15)	1.23	(1.22, 1.24)	1.24	(1.22, 1.26)	1.18	(1.16, 1.19)
FDep (CT)	1.16	(1.15, 1.16)	1.23	(1.22, 1.24)	1.25	(1.23, 1.27)	1.18	(1.16, 1.19)

CRE (CT)	1.14	(1.13, 1.14)	1.16	(1.15, 1.17)	1.22	(1.2, 1.24)	1.17	(1.16, 1.19)
Education (ACS)	1.21	(1.2, 1.22)	1.29	(1.27, 1.3)	1.27	(1.25, 1.3)	1.42	(1.4, 1.44)
Poverty index (ACS)	1.02	(1.01, 1.02)	1.09	(1.08, 1.1)	1.09	(1.07, 1.11)	1.18	(1.17, 1.2)
Occupation (ACS)	1.1	(1.09, 1.1)	1.16	(1.15, 1.17)	1.13	(1.11, 1.15)	1.21	(1.19, 1.22)

Source: American Family Cohort (AFC) data 2019-2021; American Community Survey (2005-2022); Census Numident (Q3, 2023).

Notes: Adjusted models include adjustment for age, race, ethnicity, gender, and rurality; OR = odds ratios; CI = confidence intervals; CBG = census block group; CT = census tract; ACS = American Community Survey. The Census Bureau has reviewed this data product to ensure appropriate access, use, and disclosure avoidance protection of the confidential source data used to produce this product (Data Management System (DMS) number: P-7532672, Disclosure Review Board (DRB) approval number: CBDRB-FY24-POP001-0090).

eTable 3. Similar Coefficients Plotted in Figure 2 (Adjusted Models) but Social Measures are Categorical (Quintiles)

	Hypertension		Diabetes		Chronic Kidney Disease		Mortality	
	OR	95% CI	OR	95% CI	OR	95% CI	OR	95% CI
SDI (CBG) (Ref: 5th Quintile)								
1st Quintile	0.71	(0.69, 0.73)	0.58	(0.55, 0.6)	0.54	(0.5, 0.59)	0.57	(0.53, 0.61)
2nd Quintile	0.82	(0.8, 0.84)	0.72	(0.69, 0.75)	0.68	(0.63, 0.74)	0.67	(0.63, 0.72)
3rd Quintile	0.88	(0.86, 0.91)	0.81	(0.78, 0.84)	0.85	(0.79, 0.92)	0.78	(0.73, 0.83)
4th Quintile	0.91	(0.88, 0.93)	0.88	(0.85, 0.91)	0.85	(0.79, 0.91)	0.9	(0.85, 0.96)
SDI (CT) (Ref: 5th Quintile)								
1st Quintile	0.68	(0.66, 0.71)	0.56	(0.54, 0.58)	0.55	(0.5, 0.59)	0.54	(0.5, 0.57)
2nd Quintile	0.77	(0.74, 0.79)	0.72	(0.69, 0.75)	0.65	(0.61, 0.71)	0.66	(0.62, 0.7)
3rd Quintile	0.87	(0.84, 0.89)	0.82	(0.79, 0.85)	0.88	(0.82, 0.95)	0.81	(0.76, 0.86)
4th Quintile	0.88	(0.86, 0.91)	0.89	(0.85, 0.92)	0.93	(0.86, 0.99)	0.87	(0.82, 0.92)
SVI (CBG) (Ref: 5th Quintile)								
1st Quintile	0.79	(0.77, 0.82)	0.66	(0.63, 0.69)	0.64	(0.59, 0.69)	0.63	(0.59, 0.67)
2nd Quintile	0.9	(0.87, 0.93)	0.81	(0.78, 0.85)	0.83	(0.76, 0.89)	0.77	(0.73, 0.83)
3rd Quintile	1.03	(1, 1.06)	0.9	(0.86, 0.94)	0.95	(0.88, 1.03)	0.87	(0.81, 0.92)
4th Quintile	1.04	(1.01, 1.07)	0.97	(0.93, 1.01)	1.03	(0.95, 1.11)	1.01	(0.95, 1.08)
SVI (CT) (Ref: 5th Quintile)								
1st Quintile	0.77	(0.74, 0.79)	0.66	(0.63, 0.69)	0.63	(0.58, 0.69)	0.59	(0.55, 0.63)
2nd Quintile	0.88	(0.85, 0.9)	0.77	(0.73, 0.8)	0.8	(0.74, 0.86)	0.78	(0.73, 0.83)
3rd Quintile	0.99	(0.96, 1.02)	0.94	(0.9, 0.98)	0.95	(0.88, 1.02)	0.89	(0.83, 0.95)
4th Quintile	1	(0.97, 1.03)	0.97	(0.93, 1.01)	0.96	(0.89, 1.03)	0.95	(0.89, 1.01)
ADI-GS (CBG) (Ref: 5th Quintile)								
1st Quintile	0.45	(0.43, 0.46)	0.37	(0.35, 0.39)	0.35	(0.32, 0.38)	0.46	(0.43, 0.5)
2nd Quintile	0.65	(0.63, 0.67)	0.59	(0.57, 0.62)	0.55	(0.51, 0.6)	0.56	(0.53, 0.6)
3rd Quintile	0.79	(0.77, 0.81)	0.77	(0.74, 0.79)	0.69	(0.64, 0.74)	0.68	(0.64, 0.72)
4th Quintile	0.91	(0.89, 0.94)	0.86	(0.83, 0.89)	0.92	(0.87, 0.98)	0.84	(0.8, 0.88)
ADI-GS (CT) (Ref: 5th Quintile)								
1st Quintile	0.43	(0.42, 0.45)	0.36	(0.34, 0.38)	0.35	(0.32, 0.38)	0.47	(0.44, 0.51)
2nd Quintile	0.65	(0.63, 0.67)	0.6	(0.58, 0.63)	0.53	(0.49, 0.58)	0.57	(0.53, 0.61)
3rd Quintile	0.82	(0.8, 0.84)	0.76	(0.73, 0.78)	0.7	(0.65, 0.75)	0.69	(0.65, 0.73)
4th Quintile	0.9	(0.88, 0.93)	0.87	(0.84, 0.9)	0.92	(0.86, 0.98)	0.85	(0.81, 0.9)
ADI-UW (CBG) (Ref: 5th Quintile)								
1st Quintile	0.52	(0.5, 0.54)	0.41	(0.39, 0.43)	0.38	(0.35, 0.41)	0.47	(0.44, 0.51)
2nd Quintile	0.68	(0.66, 0.7)	0.61	(0.59, 0.64)	0.53	(0.49, 0.58)	0.61	(0.57, 0.65)
3rd Quintile	0.77	(0.75, 0.79)	0.73	(0.7, 0.75)	0.67	(0.62, 0.72)	0.7	(0.67, 0.75)
4th Quintile	0.89	(0.86, 0.91)	0.85	(0.82, 0.88)	0.88	(0.82, 0.94)	0.84	(0.79, 0.89)
ADI-UW (CT) (Ref: 5th Quintile)								

1st Quintile	0.5	(0.49, 0.52)	0.41	(0.39, 0.43)	0.38	(0.35, 0.42)	0.48	(0.45, 0.52)
2nd Quintile	0.66	(0.64, 0.68)	0.59	(0.57, 0.62)	0.54	(0.5, 0.59)	0.63	(0.59, 0.67)
3rd Quintile	0.76	(0.74, 0.78)	0.72	(0.7, 0.75)	0.69	(0.64, 0.74)	0.73	(0.69, 0.77)
4th Quintile	0.88	(0.86, 0.91)	0.88	(0.85, 0.91)	0.93	(0.87, 0.99)	0.89	(0.85, 0.94)
NSS7 (CBG) (Ref: 5th Quintile)								
1st Quintile	0.62	(0.6, 0.64)	0.51	(0.48, 0.53)	0.46	(0.43, 0.5)	0.55	(0.51, 0.59)
2nd Quintile	0.74	(0.72, 0.76)	0.65	(0.62, 0.67)	0.62	(0.57, 0.66)	0.65	(0.61, 0.69)
3rd Quintile	0.8	(0.77, 0.82)	0.74	(0.72, 0.77)	0.73	(0.68, 0.79)	0.75	(0.71, 0.79)
4th Quintile	0.9	(0.88, 0.93)	0.87	(0.83, 0.9)	0.9	(0.84, 0.96)	0.87	(0.82, 0.92)
NSS7 (CT) (Ref: 5th Quintile)								
1st Quintile	0.6	(0.58, 0.62)	0.49	(0.47, 0.52)	0.48	(0.44, 0.52)	0.54	(0.5, 0.57)
2nd Quintile	0.71	(0.69, 0.73)	0.64	(0.61, 0.66)	0.6	(0.55, 0.64)	0.64	(0.6, 0.68)
3rd Quintile	0.8	(0.78, 0.82)	0.75	(0.72, 0.78)	0.77	(0.72, 0.83)	0.78	(0.74, 0.83)
4th Quintile	0.9	(0.87, 0.92)	0.87	(0.84, 0.91)	0.92	(0.86, 0.98)	0.84	(0.79, 0.89)
ICE wb-inc (CT) (Ref: 5th Quintile)								
1st Quintile	0.56	(0.54, 0.58)	0.49	(0.47, 0.51)	0.46	(0.43, 0.5)	0.55	(0.51, 0.59)
2nd Quintile	0.67	(0.65, 0.69)	0.66	(0.63, 0.69)	0.57	(0.53, 0.62)	0.66	(0.62, 0.7)
3rd Quintile	0.8	(0.78, 0.82)	0.78	(0.75, 0.81)	0.72	(0.67, 0.77)	0.77	(0.73, 0.82)
4th Quintile	0.88	(0.85, 0.9)	0.85	(0.82, 0.89)	0.83	(0.78, 0.89)	0.86	(0.82, 0.91)
ICE wpc-inc (CT) (Ref: 5th Quintile)								
1st Quintile	0.55	(0.53, 0.57)	0.45	(0.43, 0.48)	0.43	(0.4, 0.47)	0.53	(0.49, 0.57)
2nd Quintile	0.72	(0.7, 0.74)	0.67	(0.64, 0.7)	0.59	(0.54, 0.64)	0.67	(0.63, 0.72)
3rd Quintile	0.79	(0.76, 0.81)	0.75	(0.72, 0.78)	0.81	(0.75, 0.87)	0.79	(0.74, 0.84)
4th Quintile	0.87	(0.85, 0.9)	0.85	(0.81, 0.88)	0.85	(0.79, 0.92)	0.88	(0.83, 0.94)
FDep (CBG) (Ref: 5th Quintile)								
1st Quintile	0.55	(0.53, 0.56)	0.42	(0.4, 0.44)	0.38	(0.35, 0.41)	0.5	(0.47, 0.54)
2nd Quintile	0.71	(0.69, 0.73)	0.62	(0.6, 0.64)	0.57	(0.53, 0.61)	0.65	(0.61, 0.69)
3rd Quintile	0.79	(0.77, 0.81)	0.73	(0.7, 0.75)	0.69	(0.65, 0.74)	0.77	(0.72, 0.81)
4th Quintile	0.88	(0.86, 0.91)	0.83	(0.8, 0.86)	0.77	(0.72, 0.82)	0.86	(0.81, 0.91)
FDep (CT) (Ref: 5th Quintile)								
1st Quintile	0.54	(0.52, 0.55)	0.42	(0.4, 0.44)	0.38	(0.34, 0.41)	0.52	(0.48, 0.55)
2nd Quintile	0.69	(0.67, 0.71)	0.61	(0.58, 0.63)	0.57	(0.53, 0.61)	0.66	(0.62, 0.7)
3rd Quintile	0.79	(0.76, 0.81)	0.73	(0.71, 0.76)	0.66	(0.62, 0.71)	0.79	(0.74, 0.84)
4th Quintile	0.91	(0.88, 0.93)	0.86	(0.83, 0.89)	0.89	(0.83, 0.95)	0.9	(0.85, 0.95)
CRE (CT) (Ref: 5th Quintile)								
1st Quintile	0.63	(0.61, 0.64)	0.6	(0.57, 0.63)	0.47	(0.44, 0.51)	0.61	(0.57, 0.66)
2nd Quintile	0.74	(0.72, 0.76)	0.74	(0.71, 0.77)	0.66	(0.61, 0.71)	0.73	(0.69, 0.78)
3rd Quintile	0.79	(0.77, 0.81)	0.79	(0.76, 0.82)	0.65	(0.6, 0.69)	0.8	(0.75, 0.84)
4th Quintile	0.89	(0.86, 0.91)	0.87	(0.84, 0.9)	0.81	(0.76, 0.87)	0.9	(0.86, 0.96)
Education (Ref: <HS Degree)								

College +	0.71	(0.7, 0.73)	0.59	(0.57, 0.61)	0.66	(0.62, 0.7)	0.44	(0.41, 0.46)
Some College	0.96	(0.94, 0.98)	0.91	(0.88, 0.94)	0.89	(0.84, 0.95)	0.68	(0.64, 0.71)
High School	1.09	(1.06, 1.12)	1.03	(1, 1.07)	1.02	(0.96, 1.09)	0.84	(0.8, 0.89)
Poverty index (Ref: <100%)								
>400%	0.83	(0.8, 0.87)	0.67	(0.64, 0.71)	0.65	(0.58, 0.72)	0.42	(0.38, 0.45)
300-399%	0.98	(0.94, 1.02)	0.87	(0.82, 0.92)	0.88	(0.79, 0.99)	0.6	(0.55, 0.65)
200-299%	1.03	(0.99, 1.08)	0.93	(0.88, 0.99)	0.95	(0.86, 1.07)	0.73	(0.68, 0.8)
100-199%	1.04	(1, 1.09)	1.02	(0.96, 1.09)	1	(0.89, 1.12)	0.92	(0.84, 1)
Occupation (Ref: Blue collar, semi-routine)								
White collar, professional	0.81	(0.79, 0.83)	0.71	(0.69, 0.73)	0.76	(0.72, 0.8)	0.62	(0.59, 0.64)
White collar, semi-routine	0.97	(0.94, 0.99)	0.92	(0.89, 0.95)	0.96	(0.9, 1.02)	0.79	(0.75, 0.83)
Blue collar, high skill	0.99	(0.96, 1.03)	0.87	(0.83, 0.91)	0.88	(0.8, 0.96)	0.95	(0.89, 1.02)

Source: American Family Cohort (AFC) data 2019-2021; American Community Survey (2005-2022); Census Numident (Q3, 2023).

Notes: Adjusted models include adjustment for age, race, ethnicity, gender, and rurality; OR = odds ratios; CI = confidence intervals; CBG = census block group; CT = census tract; ACS = American Community Survey. The Census Bureau has reviewed this data product to ensure appropriate access, use, and disclosure avoidance protection of the confidential source data used to produce this product (Data Management System (DMS) number: P-7532672, Disclosure Review Board (DRB) approval number: CBDRB-FY24-POP001-0090).

eTable 4. Odds Ratios and 95% Confidence Intervals for Associations Between Area Measures (Deciles) of Social Risk with Health Outcomes and Mortality

	Hypertension		Diabetes		Chronic Kidney Disease		Mortality	
	OR	95% CI	OR	95% CI	OR	95% CI	OR	95% CI
SDI (CT) (Ref: 10th Decile)								
1st Decile	0.63	(0.61, 0.65)	0.43	(0.41, 0.45)	0.43	(0.38, 0.47)	0.52	(0.48, 0.57)
2nd Decile	0.74	(0.71, 0.76)	0.52	(0.49, 0.55)	0.49	(0.44, 0.54)	0.64	(0.58, 0.69)
3rd Decile	0.73	(0.7, 0.76)	0.56	(0.53, 0.59)	0.47	(0.42, 0.52)	0.71	(0.65, 0.77)
4th Decile	0.80	(0.77, 0.84)	0.65	(0.61, 0.68)	0.58	(0.53, 0.64)	0.76	(0.7, 0.82)
5th Decile	0.87	(0.84, 0.91)	0.69	(0.66, 0.73)	0.68	(0.61, 0.74)	0.89	(0.82, 0.97)
6th Decile	0.86	(0.82, 0.89)	0.69	(0.66, 0.73)	0.72	(0.66, 0.79)	0.87	(0.8, 0.94)
7th Decile	0.86	(0.83, 0.89)	0.72	(0.68, 0.76)	0.75	(0.68, 0.83)	0.89	(0.82, 0.97)
8th Decile	0.88	(0.84, 0.91)	0.79	(0.75, 0.83)	0.69	(0.63, 0.77)	0.97	(0.89, 1.05)
9th Decile	0.95	(0.91, 0.99)	0.83	(0.79, 0.87)	0.76	(0.69, 0.83)	0.95	(0.88, 1.04)
SVI (CT) (Ref: 10th Decile)								
1st Decile	0.87	(0.84, 0.91)	0.51	(0.48, 0.54)	0.58	(0.52, 0.65)	0.74	(0.68, 0.81)
2nd Decile	0.94	(0.9, 0.97)	0.60	(0.57, 0.63)	0.70	(0.63, 0.77)	0.85	(0.78, 0.93)
3rd Decile	0.99	(0.95, 1.03)	0.64	(0.61, 0.67)	0.77	(0.69, 0.85)	1.02	(0.93, 1.11)
4th Decile	1.08	(1.04, 1.12)	0.66	(0.63, 0.7)	0.79	(0.71, 0.88)	1.12	(1.02, 1.22)
5th Decile	1.15	(1.1, 1.19)	0.78	(0.74, 0.82)	0.89	(0.8, 0.98)	1.19	(1.09, 1.29)
6th Decile	1.18	(1.13, 1.23)	0.80	(0.76, 0.85)	0.97	(0.88, 1.07)	1.22	(1.12, 1.34)
7th Decile	1.14	(1.09, 1.19)	0.84	(0.8, 0.89)	0.92	(0.83, 1.02)	1.25	(1.14, 1.36)
8th Decile	1.27	(1.22, 1.32)	0.87	(0.83, 0.92)	1.02	(0.92, 1.13)	1.27	(1.16, 1.39)
9th Decile	1.12	(1.07, 1.17)	0.83	(0.79, 0.87)	0.96	(0.87, 1.07)	1.20	(1.1, 1.31)
ADI-UW (CT) (Ref: 10th Decile)								
1st Decile	0.37	(0.35, 0.38)	0.27	(0.26, 0.29)	0.31	(0.28, 0.35)	0.39	(0.36, 0.43)
2nd Decile	0.53	(0.51, 0.55)	0.45	(0.43, 0.48)	0.47	(0.42, 0.52)	0.49	(0.45, 0.53)
3rd Decile	0.63	(0.61, 0.66)	0.57	(0.55, 0.6)	0.60	(0.55, 0.67)	0.53	(0.49, 0.58)
4th Decile	0.70	(0.67, 0.72)	0.63	(0.6, 0.66)	0.59	(0.54, 0.66)	0.58	(0.54, 0.63)
5th Decile	0.79	(0.76, 0.81)	0.75	(0.71, 0.79)	0.74	(0.67, 0.82)	0.65	(0.61, 0.71)
6th Decile	0.87	(0.84, 0.9)	0.76	(0.72, 0.79)	0.81	(0.74, 0.89)	0.72	(0.67, 0.77)
7th Decile	0.90	(0.87, 0.93)	0.82	(0.78, 0.86)	0.87	(0.79, 0.95)	0.82	(0.76, 0.88)
8th Decile	0.86	(0.83, 0.89)	0.81	(0.78, 0.86)	0.98	(0.9, 1.07)	0.84	(0.78, 0.91)
9th Decile	0.91	(0.88, 0.95)	0.88	(0.84, 0.92)	1.00	(0.92, 1.09)	0.91	(0.85, 0.98)
ADI-GS (CT) (Ref: 10th Decile)								
1st Decile	0.44	(0.42, 0.46)	0.31	(0.29, 0.33)	0.27	(0.24, 0.3)	0.39	(0.35, 0.42)
2nd Decile	0.57	(0.55, 0.59)	0.45	(0.43, 0.48)	0.44	(0.4, 0.49)	0.53	(0.49, 0.58)
3rd Decile	0.63	(0.61, 0.65)	0.52	(0.49, 0.55)	0.48	(0.44, 0.54)	0.61	(0.57, 0.67)
4th Decile	0.66	(0.64, 0.69)	0.55	(0.53, 0.58)	0.52	(0.48, 0.58)	0.65	(0.6, 0.7)
5th Decile	0.73	(0.7, 0.75)	0.65	(0.62, 0.69)	0.57	(0.52, 0.63)	0.70	(0.65, 0.76)

6th Decile	0.77	(0.74, 0.79)	0.64	(0.61, 0.67)	0.62	(0.57, 0.68)	0.77	(0.71, 0.83)
7th Decile	0.82	(0.79, 0.85)	0.72	(0.69, 0.76)	0.69	(0.63, 0.76)	0.86	(0.8, 0.93)
8th Decile	0.89	(0.85, 0.92)	0.81	(0.77, 0.85)	0.89	(0.81, 0.97)	0.92	(0.85, 0.99)
9th Decile	0.92	(0.89, 0.96)	0.84	(0.8, 0.88)	0.81	(0.74, 0.88)	0.90	(0.83, 0.97)
NSS7 (CT) (Ref: 10th Decile)								
1st Decile	0.56	(0.53, 0.58)	0.38	(0.36, 0.4)	0.37	(0.33, 0.41)	0.50	(0.46, 0.55)
2nd Decile	0.63	(0.61, 0.65)	0.47	(0.44, 0.49)	0.49	(0.44, 0.54)	0.62	(0.57, 0.67)
3rd Decile	0.66	(0.63, 0.68)	0.51	(0.48, 0.54)	0.50	(0.46, 0.56)	0.64	(0.59, 0.7)
4th Decile	0.73	(0.7, 0.75)	0.58	(0.55, 0.61)	0.51	(0.46, 0.57)	0.73	(0.67, 0.79)
5th Decile	0.74	(0.71, 0.77)	0.60	(0.57, 0.63)	0.57	(0.51, 0.62)	0.83	(0.76, 0.9)
6th Decile	0.81	(0.78, 0.84)	0.68	(0.65, 0.71)	0.73	(0.66, 0.8)	0.86	(0.79, 0.93)
7th Decile	0.84	(0.81, 0.87)	0.71	(0.68, 0.75)	0.72	(0.65, 0.79)	0.85	(0.79, 0.93)
8th Decile	0.89	(0.86, 0.92)	0.78	(0.74, 0.82)	0.80	(0.73, 0.88)	0.93	(0.86, 1.01)
9th Decile	0.93	(0.9, 0.97)	0.84	(0.8, 0.88)	0.85	(0.78, 0.93)	0.98	(0.91, 1.07)
ICE – INC BW (CT) (Ref: 10th Decile)								
1st Decile	0.43	(0.42, 0.45)	0.36	(0.34, 0.38)	0.31	(0.28, 0.34)	0.42	(0.39, 0.46)
2nd Decile	0.51	(0.49, 0.53)	0.48	(0.46, 0.51)	0.44	(0.4, 0.48)	0.56	(0.51, 0.6)
3rd Decile	0.52	(0.5, 0.54)	0.51	(0.49, 0.54)	0.43	(0.39, 0.48)	0.59	(0.54, 0.64)
4th Decile	0.58	(0.55, 0.6)	0.61	(0.58, 0.64)	0.45	(0.41, 0.49)	0.64	(0.59, 0.7)
5th Decile	0.65	(0.63, 0.67)	0.65	(0.62, 0.68)	0.54	(0.49, 0.59)	0.71	(0.66, 0.77)
6th Decile	0.64	(0.62, 0.66)	0.66	(0.62, 0.69)	0.56	(0.51, 0.61)	0.74	(0.68, 0.79)
7th Decile	0.67	(0.65, 0.69)	0.67	(0.64, 0.7)	0.60	(0.55, 0.66)	0.81	(0.75, 0.87)
8th Decile	0.75	(0.72, 0.78)	0.74	(0.71, 0.78)	0.64	(0.58, 0.69)	0.86	(0.79, 0.92)
9th Decile	0.79	(0.77, 0.82)	0.81	(0.78, 0.85)	0.80	(0.74, 0.87)	0.91	(0.84, 0.98)
ICE – INC WNH (CT) (Ref: 10th Decile)								
1st Decile	0.46	(0.44, 0.48)	0.31	(0.3, 0.33)	0.27	(0.24, 0.3)	0.48	(0.44, 0.52)
2nd Decile	0.55	(0.53, 0.57)	0.42	(0.4, 0.44)	0.40	(0.36, 0.44)	0.64	(0.59, 0.7)
3rd Decile	0.63	(0.61, 0.65)	0.51	(0.48, 0.54)	0.39	(0.35, 0.43)	0.74	(0.68, 0.8)
4th Decile	0.68	(0.66, 0.71)	0.56	(0.53, 0.59)	0.45	(0.41, 0.49)	0.79	(0.72, 0.85)
5th Decile	0.69	(0.67, 0.72)	0.56	(0.54, 0.59)	0.49	(0.45, 0.54)	0.87	(0.8, 0.94)
6th Decile	0.74	(0.71, 0.77)	0.61	(0.58, 0.64)	0.62	(0.56, 0.67)	0.95	(0.88, 1.03)
7th Decile	0.79	(0.76, 0.82)	0.67	(0.64, 0.7)	0.65	(0.59, 0.71)	0.98	(0.91, 1.07)
8th Decile	0.76	(0.74, 0.79)	0.70	(0.66, 0.73)	0.58	(0.53, 0.63)	0.99	(0.91, 1.07)
9th Decile	0.80	(0.77, 0.83)	0.79	(0.75, 0.83)	0.64	(0.59, 0.71)	0.95	(0.88, 1.04)
FDep (CT) (Ref: 10th Decile)								
1st Decile	0.51	(0.49, 0.53)	0.31	(0.3, 0.33)	0.30	(0.27, 0.34)	0.43	(0.39, 0.47)
2nd Decile	0.63	(0.61, 0.66)	0.45	(0.42, 0.47)	0.44	(0.39, 0.48)	0.58	(0.53, 0.63)
3rd Decile	0.71	(0.69, 0.74)	0.52	(0.49, 0.54)	0.53	(0.48, 0.59)	0.66	(0.61, 0.72)
4th Decile	0.76	(0.73, 0.79)	0.56	(0.53, 0.59)	0.55	(0.5, 0.61)	0.70	(0.64, 0.76)
5th Decile	0.83	(0.8, 0.87)	0.64	(0.61, 0.67)	0.61	(0.56, 0.68)	0.86	(0.79, 0.93)

6th Decile	0.85	(0.82, 0.88)	0.65	(0.62, 0.69)	0.62	(0.56, 0.68)	0.83	(0.77, 0.9)
7th Decile	0.93	(0.9, 0.97)	0.73	(0.69, 0.76)	0.80	(0.73, 0.88)	0.94	(0.87, 1.02)
8th Decile	0.99	(0.95, 1.03)	0.79	(0.75, 0.83)	0.79	(0.72, 0.87)	0.94	(0.87, 1.02)
9th Decile	1.05	(1.01, 1.09)	0.87	(0.83, 0.91)	0.91	(0.83, 0.99)	0.95	(0.87, 1.03)
CRE (CT) (Ref: 10th Decile)								
1st Decile	0.55	(0.53, 0.57)	0.47	(0.45, 0.5)	0.38	(0.34, 0.42)	0.44	(0.41, 0.48)
2nd Decile	0.63	(0.61, 0.65)	0.56	(0.53, 0.59)	0.44	(0.4, 0.49)	0.59	(0.54, 0.64)
3rd Decile	0.69	(0.66, 0.71)	0.63	(0.6, 0.66)	0.55	(0.5, 0.6)	0.63	(0.58, 0.68)
4th Decile	0.71	(0.69, 0.74)	0.66	(0.63, 0.7)	0.57	(0.52, 0.63)	0.67	(0.62, 0.72)
5th Decile	0.74	(0.72, 0.77)	0.68	(0.65, 0.72)	0.54	(0.49, 0.59)	0.72	(0.67, 0.78)
6th Decile	0.79	(0.77, 0.82)	0.72	(0.69, 0.76)	0.60	(0.54, 0.65)	0.78	(0.72, 0.84)
7th Decile	0.83	(0.8, 0.86)	0.76	(0.73, 0.8)	0.69	(0.63, 0.76)	0.87	(0.8, 0.93)
8th Decile	0.89	(0.86, 0.93)	0.83	(0.79, 0.87)	0.75	(0.69, 0.82)	0.88	(0.81, 0.94)
9th Decile	0.99	(0.96, 1.03)	0.94	(0.89, 0.98)	0.92	(0.84, 1)	0.97	(0.9, 1.04)

Source: American Family Cohort (AFC) data 2019-2021; American Community Survey (2005-2022); Census Numident (Q3, 2023).

Notes: Models are unadjusted; OR = odds ratios; CI = confidence intervals; CT = census tract. The Census Bureau has reviewed this data product to ensure appropriate access, use, and disclosure avoidance protection of the confidential source data used to produce this product (Data Management System (DMS) number: P-7532672, Disclosure Review Board (DRB) approval number: CBDRB-FY25-POP001-0068).

eTable 5. Coefficients Plotted in Figure 3 (Race), Including Smaller Racial Groups (AIAN, NHPI, Two or More Races)

Hypertension

	White		Black		AIAN		Asian		NHPI		Two or more races		Missing race	
	OR	95% CI	OR	95% CI	OR	95% CI	OR	95% CI	OR	95% CI	OR	95% CI	OR	95% CI
SDI (CBG)	1.07	(1.06, 1.08)	1.04	(1.01, 1.06)	1.06	(0.96, 1.17)	1.15	(1.1, 1.19)	1.03	(0.89, 1.18)	1.05	(0.76, 1.45)	1.05	(1.03, 1.07)
SDI (CT)	1.08	(1.08, 1.09)	1.04	(1.02, 1.06)	1.17	(1.05, 1.29)	1.17	(1.13, 1.22)	1.08	(0.94, 1.23)	1.12	(0.8, 1.57)	1.05	(1.04, 1.07)
SVI (CBG)	1.05	(1.05, 1.06)	1	(0.98, 1.03)	1.16	(1.04, 1.29)	1.16	(1.12, 1.21)	0.99	(0.87, 1.14)	0.8	(0.59, 1.09)	1.04	(1.02, 1.06)
SVI (CT)	1.06	(1.06, 1.07)	1	(0.97, 1.02)	1.21	(1.08, 1.35)	1.17	(1.13, 1.22)	1.01	(0.88, 1.15)	0.95	(0.69, 1.31)	1.04	(1.03, 1.06)
ADI-GS (CBG)	1.15	(1.14, 1.16)	1.1	(1.08, 1.13)	1.18	(1.07, 1.3)	1.29	(1.24, 1.35)	1.14	(0.98, 1.31)	1	(0.68, 1.45)	1.14	(1.12, 1.16)
ADI-GS (CT)	1.16	(1.15, 1.17)	1.1	(1.07, 1.12)	1.26	(1.14, 1.39)	1.33	(1.27, 1.38)	1.12	(0.97, 1.3)	1.24	(0.84, 1.84)	1.13	(1.12, 1.15)
ADI-UW (CBG)	1.16	(1.16, 1.17)	1.13	(1.1, 1.16)	1.12	(1.02, 1.24)	1.21	(1.16, 1.27)	1.24	(1.07, 1.44)	1.08	(0.72, 1.61)	1.2	(1.19, 1.22)
ADI-UW (CT)	1.16	(1.16, 1.17)	1.13	(1.1, 1.15)	1.15	(1.04, 1.27)	1.23	(1.17, 1.29)	1.25	(1.08, 1.45)	1.25	(0.8, 1.95)	1.2	(1.18, 1.21)
NSS7 (CBG)	1.1	(1.1, 1.11)	1.07	(1.05, 1.1)	1.14	(1.03, 1.26)	1.24	(1.19, 1.29)	1.11	(0.96, 1.27)	0.98	(0.71, 1.37)	1.09	(1.07, 1.11)
NSS7 (CT)	1.12	(1.11, 1.13)	1.08	(1.06, 1.1)	1.26	(1.14, 1.39)	1.27	(1.21, 1.32)	1.09	(0.96, 1.25)	1.08	(0.76, 1.54)	1.1	(1.08, 1.11)
ICE WB+INC (CT)	1.13	(1.13, 1.14)	1.13	(1.11, 1.16)	1.18	(1.07, 1.31)	1.21	(1.16, 1.27)	1.18	(1.03, 1.36)	1.15	(0.85, 1.55)	1.15	(1.13, 1.17)
ICE WPC+INC (CT)	1.13	(1.12, 1.14)	1.1	(1.07, 1.12)	1.26	(1.14, 1.4)	1.29	(1.24, 1.34)	1.11	(0.98, 1.27)	1.1	(0.8, 1.51)	1.11	(1.1, 1.13)
FDep (CBG)	1.14	(1.14, 1.15)	1.1	(1.08, 1.12)	1.19	(1.08, 1.32)	1.29	(1.23, 1.34)	1.1	(0.95, 1.27)	1.09	(0.78, 1.54)	1.1	(1.09, 1.12)
FDep (CT)	1.16	(1.15, 1.16)	1.1	(1.08, 1.12)	1.23	(1.1, 1.37)	1.3	(1.25, 1.36)	1.08	(0.94, 1.25)	1.3	(0.88, 1.9)	1.1	(1.09, 1.12)
CRE (CT)	1.13	(1.12, 1.13)	1.09	(1.07, 1.11)	1.15	(1.04, 1.26)	1.23	(1.17, 1.28)	1.02	(0.89, 1.17)	1.2	(0.87, 1.65)	1.12	(1.1, 1.13)
Education (ACS)	1.22	(1.21, 1.23)	1.13	(1.1, 1.17)	1.03	(0.91, 1.17)	1.25	(1.19, 1.32)	1.12	(0.94, 1.34)	0.88	(0.58, 1.32)	1.19	(1.17, 1.22)
Poverty index (ACS)	1.02	(1.01, 1.02)	0.95	(0.93, 0.97)	0.92	(0.84, 1.01)	1.14	(1.09, 1.19)	0.94	(0.81, 1.09)	1.01	(0.7, 1.45)	1.01	(0.99, 1.03)
Occupation (ACS)	1.1	(1.09, 1.11)	1.03	(1.01, 1.06)	1.03	(0.93, 1.14)	1.21	(1.16, 1.26)	1.07	(0.92, 1.24)	1.29	(0.91, 1.84)	1.08	(1.07, 1.1)

Diabetes

	White		Black		AIAN		Asian		NHPI		Two or more races		Missing	
	OR	95% CI	OR	95% CI	OR	95% CI	OR	95% CI	OR	95% CI	OR	95% CI	OR	95% CI
SDI (CBG)	1.14	(1.13, 1.15)	1.05	(1.02, 1.08)	1.31	(1.15, 1.49)	1.12	(1.06, 1.18)	1.24	(1.03, 1.49)	1.37	(0.94, 2)	1.13	(1.11, 1.16)
SDI (CT)	1.16	(1.14, 1.17)	1.06	(1.03, 1.09)	1.32	(1.15, 1.51)	1.14	(1.08, 1.2)	1.28	(1.07, 1.53)	1.28	(0.88, 1.86)	1.15	(1.13, 1.18)
SVI (CBG)	1.12	(1.11, 1.14)	1.01	(0.98, 1.04)	1.44	(1.25, 1.67)	1.15	(1.09, 1.21)	1.13	(0.95, 1.34)	1.13	(0.79, 1.6)	1.14	(1.11, 1.16)
SVI (CT)	1.13	(1.12, 1.14)	1.03	(0.99, 1.06)	1.35	(1.17, 1.55)	1.16	(1.1, 1.22)	1.17	(0.99, 1.39)	1.2	(0.83, 1.72)	1.16	(1.13, 1.18)
ADI-GS (CBG)	1.22	(1.21, 1.23)	1.13	(1.1, 1.16)	1.37	(1.19, 1.56)	1.24	(1.17, 1.31)	1.21	(1, 1.45)	1.12	(0.73, 1.72)	1.23	(1.2, 1.26)
ADI-GS (CT)	1.22	(1.21, 1.23)	1.13	(1.1, 1.16)	1.39	(1.22, 1.59)	1.25	(1.18, 1.32)	1.37	(1.14, 1.65)	1.42	(0.91, 2.19)	1.23	(1.21, 1.26)
ADI-UW (CBG)	1.22	(1.21, 1.23)	1.16	(1.12, 1.19)	1.25	(1.1, 1.42)	1.24	(1.17, 1.32)	1.17	(0.97, 1.41)	1.08	(0.68, 1.72)	1.25	(1.22, 1.28)
ADI-UW (CT)	1.21	(1.2, 1.22)	1.16	(1.12, 1.19)	1.24	(1.09, 1.41)	1.25	(1.18, 1.33)	1.25	(1.04, 1.51)	1.27	(0.78, 2.09)	1.24	(1.22, 1.27)
NSS7 (CBG)	1.18	(1.17, 1.19)	1.09	(1.06, 1.12)	1.38	(1.2, 1.57)	1.2	(1.14, 1.27)	1.23	(1.03, 1.47)	1.32	(0.91, 1.93)	1.19	(1.16, 1.22)
NSS7 (CT)	1.19	(1.18, 1.2)	1.11	(1.08, 1.14)	1.4	(1.23, 1.6)	1.21	(1.15, 1.28)	1.3	(1.08, 1.55)	1.35	(0.9, 2.02)	1.2	(1.18, 1.23)
ICE WB+INC (CT)	1.16	(1.15, 1.17)	1.1	(1.07, 1.13)	1.34	(1.17, 1.54)	1.2	(1.14, 1.27)	1.18	(0.99, 1.41)	1.25	(0.89, 1.76)	1.2	(1.17, 1.22)
ICE WPC+INC (CT)	1.21	(1.19, 1.22)	1.11	(1.08, 1.15)	1.37	(1.19, 1.57)	1.23	(1.17, 1.29)	1.27	(1.07, 1.51)	1.24	(0.87, 1.77)	1.23	(1.21, 1.26)
FDep (CBG)	1.22	(1.21, 1.23)	1.14	(1.1, 1.17)	1.33	(1.16, 1.52)	1.25	(1.19, 1.31)	1.22	(1.02, 1.47)	1.32	(0.89, 1.95)	1.23	(1.2, 1.25)
FDep (CT)	1.23	(1.22, 1.24)	1.14	(1.1, 1.17)	1.31	(1.14, 1.51)	1.23	(1.17, 1.3)	1.3	(1.08, 1.56)	1.54	(1, 2.37)	1.22	(1.2, 1.25)
CRE (CT)	1.15	(1.14, 1.16)	1.1	(1.07, 1.13)	1.36	(1.19, 1.55)	1.14	(1.08, 1.21)	1.03	(0.87, 1.23)	1.46	(1.02, 2.1)	1.16	(1.14, 1.19)

Education (ACS)	1.29	(1.27, 1.3)	1.19	(1.15, 1.24)	1.14	(0.98, 1.34)	1.28	(1.2, 1.36)	1.58	(1.26, 1.98)	1.82	(1.09, 3.04)	1.34	(1.3, 1.38)
Poverty index (ACS)	1.09	(1.07, 1.1)	1	(0.97, 1.03)	1.05	(0.93, 1.18)	1.11	(1.04, 1.18)	1.11	(0.92, 1.33)	1.04	(0.69, 1.59)	1.1	(1.08, 1.13)
Occupation (ACS)	1.16	(1.15, 1.17)	1.1	(1.07, 1.13)	1.03	(0.9, 1.17)	1.2	(1.13, 1.27)	1.26	(1.04, 1.52)	0.94	(0.62, 1.41)	1.2	(1.17, 1.23)
Chronic Kidney Disease														
	White		Black		AIAN		Asian		NHPI		Two or more races		Missing	
	OR	95% CI	OR	95% CI	OR	95% CI	OR	95% CI	OR	95% CI	OR	95% CI	OR	95% CI
SDI (CBG)	1.12	(1.1, 1.14)	1.14	(1.08, 1.19)	0.95	(0.72, 1.26)	1.15	(1.03, 1.29)	1.21	(0.81, 1.79)	2.31	(1, 5.33)	1.13	(1.08, 1.19)
SDI (CT)	1.14	(1.12, 1.17)	1.13	(1.08, 1.18)	0.86	(0.64, 1.16)	1.23	(1.1, 1.38)	1.37	(0.92, 2.04)	3.1	(1.16, 8.27)	1.11	(1.06, 1.16)
SVI (CBG)	1.1	(1.08, 1.12)	1.04	(0.99, 1.09)	1.09	(0.79, 1.5)	1.19	(1.06, 1.34)	1.39	(0.92, 2.1)	1.87	(0.91, 3.82)	1.13	(1.08, 1.18)
SVI (CT)	1.11	(1.09, 1.13)	1.03	(0.98, 1.08)	1.02	(0.75, 1.41)	1.25	(1.1, 1.41)	1.27	(0.86, 1.88)	1.78	(0.9, 3.53)	1.1	(1.05, 1.15)
ADI-GS (CBG)	1.22	(1.2, 1.24)	1.21	(1.15, 1.27)	1.04	(0.78, 1.38)	1.31	(1.17, 1.47)	1.53	(1.01, 2.33)	2.12	(0.96, 4.69)	1.23	(1.18, 1.29)
ADI-GS (CT)	1.22	(1.2, 1.25)	1.21	(1.15, 1.26)	1.03	(0.77, 1.36)	1.36	(1.22, 1.53)	1.52	(1.01, 2.3)	2.83	(1.12, 7.15)	1.21	(1.16, 1.27)
ADI-UW (CBG)	1.2	(1.17, 1.22)	1.26	(1.2, 1.33)	1.19	(0.88, 1.61)	1.19	(1.04, 1.35)	1.45	(0.97, 2.14)	1.72	(0.77, 3.83)	1.28	(1.23, 1.34)
ADI-UW (CT)	1.19	(1.17, 1.21)	1.29	(1.22, 1.35)	1.07	(0.8, 1.44)	1.18	(1.04, 1.34)	1.57	(1.05, 2.34)	1.71	(0.71, 4.12)	1.26	(1.21, 1.32)
NSS7 (CBG)	1.17	(1.15, 1.2)	1.17	(1.12, 1.23)	0.96	(0.72, 1.28)	1.29	(1.15, 1.45)	1.78	(1.12, 2.84)	2.36	(1.04, 5.35)	1.19	(1.13, 1.24)
NSS7 (CT)	1.18	(1.16, 1.21)	1.19	(1.14, 1.25)	0.94	(0.71, 1.24)	1.28	(1.14, 1.44)	1.44	(0.96, 2.16)	3.54	(1.27, 9.87)	1.15	(1.1, 1.21)
ICE WB+INC (CT)	1.18	(1.16, 1.21)	1.23	(1.16, 1.3)	1.11	(0.82, 1.5)	1.14	(1.02, 1.29)	1.68	(1.07, 2.66)	1.82	(0.89, 3.72)	1.21	(1.15, 1.27)
ICE WPC+INC (CT)	1.2	(1.17, 1.22)	1.22	(1.15, 1.28)	0.99	(0.74, 1.32)	1.34	(1.19, 1.51)	1.49	(0.99, 2.26)	2.37	(1.05, 5.32)	1.22	(1.17, 1.28)
FDep (CBG)	1.22	(1.19, 1.24)	1.17	(1.11, 1.22)	1.17	(0.86, 1.59)	1.35	(1.21, 1.51)	2.08	(1.28, 3.39)	2.03	(0.92, 4.49)	1.21	(1.16, 1.27)
FDep (CT)	1.23	(1.21, 1.26)	1.19	(1.14, 1.25)	1.12	(0.82, 1.54)	1.4	(1.25, 1.56)	1.6	(1.05, 2.43)	2.53	(1.03, 6.17)	1.19	(1.13, 1.24)
CRE (CT)	1.19	(1.17, 1.21)	1.21	(1.16, 1.27)	0.97	(0.73, 1.29)	1.26	(1.12, 1.41)	0.83	(0.56, 1.23)	1.38	(0.71, 2.65)	1.21	(1.15, 1.26)
Education (ACS)	1.29	(1.27, 1.32)	1.16	(1.09, 1.22)	1.05	(0.72, 1.51)	1.34	(1.18, 1.53)	2.48	(1.41, 4.35)	0.76	(0.29, 2.01)	1.18	(1.12, 1.25)
Poverty index (ACS)	1.1	(1.07, 1.12)	0.96	(0.92, 1.01)	1.1	(0.84, 1.45)	1.18	(1.04, 1.34)	1.25	(0.85, 1.83)	0.86	(0.35, 2.1)	1.07	(1.02, 1.12)
Occupation (ACS)	1.13	(1.11, 1.15)	1.06	(1.01, 1.11)	1.24	(0.91, 1.69)	1.27	(1.12, 1.43)	1.45	(0.93, 2.26)	1.02	(0.47, 2.2)	1.13	(1.08, 1.19)
Mortality														
	White		Black		AIAN		Asian		NHPI		Two or more races		Missing	
	OR	95% CI	OR	95% CI	OR	95% CI	OR	95% CI	OR	95% CI	OR	95% CI	OR	95% CI
SDI (CBG)	1.14	(1.12, 1.15)	1.17	(1.11, 1.24)	1.13	(0.91, 1.4)	1.27	(1.11, 1.45)	0.95	(0.7, 1.3)			1.11	(1.07, 1.14)
SDI (CT)	1.15	(1.13, 1.17)	1.16	(1.1, 1.23)	1.33	(1.05, 1.7)	1.29	(1.13, 1.48)	1.09	(0.81, 1.47)			1.11	(1.07, 1.15)
SVI (CBG)	1.11	(1.1, 1.13)	1.08	(1.02, 1.14)	1.06	(0.84, 1.33)	1.31	(1.13, 1.51)	1.06	(0.78, 1.43)			1.07	(1.03, 1.1)
SVI (CT)	1.12	(1.1, 1.14)	1.06	(1, 1.12)	1.25	(0.98, 1.6)	1.28	(1.11, 1.48)	1.02	(0.76, 1.37)			1.04	(1.01, 1.08)
ADI-GS (CBG)	1.19	(1.17, 1.21)	1.26	(1.19, 1.33)	1.17	(0.94, 1.45)	1.39	(1.22, 1.59)	1.34	(0.97, 1.85)			1.21	(1.17, 1.26)
ADI-GS (CT)	1.18	(1.16, 1.2)	1.24	(1.17, 1.31)	1.21	(0.97, 1.51)	1.39	(1.22, 1.59)	1.41	(1.01, 1.95)			1.21	(1.17, 1.25)
ADI-UW (CBG)	1.19	(1.17, 1.21)	1.28	(1.2, 1.35)	1.3	(1.03, 1.64)	1.15	(0.99, 1.34)	1.34	(0.97, 1.84)			1.29	(1.25, 1.33)
ADI-UW (CT)	1.18	(1.17, 1.2)	1.23	(1.16, 1.31)	1.46	(1.14, 1.87)	1.16	(1, 1.35)	1.37	(0.99, 1.89)			1.26	(1.22, 1.3)
NSS7 (CBG)	1.15	(1.13, 1.16)	1.17	(1.11, 1.24)	1.05	(0.85, 1.29)	1.34	(1.17, 1.54)	1.06	(0.78, 1.44)			1.16	(1.12, 1.2)
NSS7 (CT)	1.15	(1.14, 1.17)	1.17	(1.11, 1.24)	1.23	(0.98, 1.53)	1.34	(1.18, 1.54)	1.09	(0.8, 1.47)			1.15	(1.12, 1.19)
ICE WB+INC (CT)	1.17	(1.16, 1.19)	1.21	(1.13, 1.29)	1.13	(0.9, 1.41)	1.2	(1.04, 1.38)	1.29	(0.94, 1.78)			1.21	(1.16, 1.25)
ICE WPC+INC (CT)	1.17	(1.15, 1.19)	1.17	(1.09, 1.24)	1.32	(1.04, 1.67)	1.35	(1.18, 1.55)	1.21	(0.9, 1.64)			1.12	(1.08, 1.15)
FDep (CBG)	1.17	(1.15, 1.19)	1.24	(1.17, 1.31)	1.01	(0.81, 1.25)	1.39	(1.22, 1.57)	1.07	(0.78, 1.48)			1.17	(1.13, 1.21)
FDep (CT)	1.18	(1.16, 1.19)	1.22	(1.15, 1.29)	1.21	(0.95, 1.52)	1.43	(1.26, 1.62)	1.05	(0.77, 1.44)			1.15	(1.12, 1.19)

CRE (CT)	1.16	(1.14, 1.17)	1.22	(1.15, 1.28)	1.25	(0.99, 1.56)	1.11	(0.97, 1.27)	1.2	(0.89, 1.61)			1.22	(1.18, 1.27)
Education (ACS)	1.44	(1.41, 1.46)	1.42	(1.33, 1.52)	1.13	(0.86, 1.47)	1.35	(1.16, 1.57)	1.62	(1.09, 2.4)			1.34	(1.28, 1.39)
Poverty index (ACS)	1.2	(1.18, 1.22)	1.12	(1.06, 1.17)	1.1	(0.9, 1.34)	1.1	(0.95, 1.28)	1.27	(0.94, 1.72)			1.17	(1.13, 1.21)
Occupation (ACS)	1.21	(1.19, 1.23)	1.25	(1.18, 1.33)	1.19	(0.95, 1.49)	1.23	(1.07, 1.42)	1.27	(0.9, 1.77)			1.19	(1.14, 1.23)

Source: American Family Cohort (AFC) data 2019-2021; American Community Survey (2005-2022); Census Numident (Q3, 2023).

Notes: OR = odds ratios; CI = confidence intervals; AIAN = American Indian or Alaska Native; NHPI = Native Hawaiian or Pacific Islander; CBG = census block group; CT = census tract; ACS = American Community Survey. The Census Bureau has reviewed this data product to ensure appropriate access, use, and disclosure avoidance protection of the confidential source data used to produce this product (Data Management System (DMS) number: P-7532672, Disclosure Review Board (DRB) approval number: CBDRB-FY24-POP001-0090).

eTable 6. Coefficients Plotted in Figure 4 (Ethnicity)

	Hypertension					
	Not Hispanic		Hispanic		Missing ethnicity	
	B	95% CI	B	95% CI	B	95% CI
SDI (CBG)	1.09	(1.09, 1.1)	1.05	(1.03, 1.08)	1.08	(1.06, 1.09)
SDI (CT)	1.11	(1.1, 1.12)	1.06	(1.03, 1.08)	1.08	(1.07, 1.09)
SVI (CBG)	1.07	(1.06, 1.08)	1.06	(1.03, 1.08)	1.05	(1.03, 1.06)
SVI (CT)	1.08	(1.08, 1.09)	1.05	(1.03, 1.08)	1.06	(1.04, 1.07)
ADI-GS (CBG)	1.18	(1.17, 1.19)	1.14	(1.12, 1.17)	1.15	(1.13, 1.16)
ADI-GS (CT)	1.19	(1.18, 1.2)	1.14	(1.12, 1.17)	1.15	(1.13, 1.16)
ADI-UW (CBG)	1.18	(1.17, 1.18)	1.23	(1.21, 1.26)	1.2	(1.18, 1.21)
ADI-UW (CT)	1.18	(1.17, 1.18)	1.23	(1.21, 1.26)	1.2	(1.18, 1.21)
NSS7 (CBG)	1.13	(1.13, 1.14)	1.09	(1.07, 1.12)	1.11	(1.1, 1.12)
NSS7 (CT)	1.15	(1.14, 1.16)	1.1	(1.07, 1.13)	1.12	(1.11, 1.13)
ICE WB+INC (CT)	1.17	(1.17, 1.18)	1.01	(0.99, 1.04)	1.16	(1.14, 1.17)
ICE WPC+INC (CT)	1.17	(1.17, 1.18)	1.13	(1.1, 1.16)	1.14	(1.13, 1.15)
FDep (CBG)	1.18	(1.17, 1.18)	1.1	(1.07, 1.12)	1.13	(1.11, 1.14)
FDep (CT)	1.19	(1.18, 1.2)	1.09	(1.07, 1.12)	1.13	(1.12, 1.14)
CRE (CT)	1.15	(1.14, 1.16)	1.13	(1.11, 1.16)	1.11	(1.1, 1.13)
Education (ACS)	1.23	(1.22, 1.24)	1.2	(1.16, 1.23)	1.2	(1.18, 1.21)
Poverty index (ACS)	1.03	(1.02, 1.03)	1.02	(0.99, 1.04)	1.02	(1.01, 1.04)
Occupation (ACS)	1.11	(1.1, 1.12)	1.1	(1.07, 1.13)	1.09	(1.07, 1.1)

	Diabetes					
	Not Hispanic		Hispanic		Missing	
	B	95% CI	B	95% CI	B	95% CI
SDI (CBG)	1.15	(1.14, 1.16)	1.12	(1.09, 1.15)	1.12	(1.1, 1.14)
SDI (CT)	1.16	(1.15, 1.18)	1.15	(1.11, 1.18)	1.13	(1.11, 1.15)
SVI (CBG)	1.12	(1.11, 1.13)	1.09	(1.06, 1.12)	1.1	(1.08, 1.12)
SVI (CT)	1.13	(1.12, 1.14)	1.13	(1.1, 1.16)	1.1	(1.09, 1.12)
ADI-GS (CBG)	1.23	(1.22, 1.24)	1.19	(1.16, 1.23)	1.2	(1.18, 1.23)
ADI-GS (CT)	1.24	(1.22, 1.25)	1.21	(1.18, 1.25)	1.2	(1.18, 1.22)
ADI-UW (CBG)	1.23	(1.22, 1.24)	1.23	(1.2, 1.26)	1.24	(1.22, 1.26)
ADI-UW (CT)	1.22	(1.21, 1.24)	1.23	(1.2, 1.26)	1.23	(1.21, 1.25)
NSS7 (CBG)	1.19	(1.18, 1.2)	1.16	(1.12, 1.19)	1.17	(1.15, 1.19)
NSS7 (CT)	1.2	(1.19, 1.22)	1.17	(1.14, 1.21)	1.18	(1.16, 1.2)
ICE WB+INC (CT)	1.22	(1.2, 1.23)	1.04	(1.01, 1.07)	1.19	(1.17, 1.21)
ICE WPC+INC (CT)	1.22	(1.21, 1.24)	1.19	(1.16, 1.23)	1.2	(1.18, 1.22)
FDep (CBG)	1.23	(1.22, 1.25)	1.17	(1.14, 1.2)	1.2	(1.18, 1.22)
FDep (CT)	1.24	(1.23, 1.26)	1.17	(1.14, 1.2)	1.19	(1.17, 1.21)
CRE (CT)	1.16	(1.15, 1.17)	1.15	(1.12, 1.18)	1.13	(1.11, 1.15)
Education (ACS)	1.28	(1.26, 1.29)	1.33	(1.28, 1.38)	1.26	(1.23, 1.28)
Poverty index (ACS)	1.09	(1.07, 1.1)	1.06	(1.03, 1.09)	1.08	(1.06, 1.1)
Occupation (ACS)	1.16	(1.15, 1.17)	1.17	(1.14, 1.21)	1.14	(1.12, 1.16)

	Chronic Kidney Disease					
	Not Hispanic		Hispanic		Missing	
	B	95% CI	B	95% CI	B	95% CI
SDI (CBG)	1.18	(1.16, 1.2)	1.13	(1.06, 1.21)	1.11	(1.08, 1.15)
SDI (CT)	1.21	(1.18, 1.23)	1.15	(1.08, 1.23)	1.11	(1.08, 1.15)
SVI (CBG)	1.14	(1.12, 1.16)	1.09	(1.02, 1.17)	1.09	(1.06, 1.12)
SVI (CT)	1.15	(1.12, 1.17)	1.1	(1.03, 1.19)	1.08	(1.05, 1.12)
ADI-GS (CBG)	1.28	(1.25, 1.3)	1.24	(1.16, 1.32)	1.2	(1.16, 1.24)
ADI-GS (CT)	1.28	(1.26, 1.31)	1.25	(1.17, 1.33)	1.19	(1.16, 1.23)
ADI-UW (CBG)	1.24	(1.22, 1.27)	1.27	(1.2, 1.35)	1.24	(1.2, 1.28)
ADI-UW (CT)	1.24	(1.22, 1.27)	1.25	(1.18, 1.33)	1.23	(1.2, 1.27)
NSS7 (CBG)	1.23	(1.21, 1.26)	1.22	(1.14, 1.31)	1.17	(1.13, 1.2)

NSS7 (CT)	1.25	(1.22, 1.27)	1.21	(1.13, 1.3)	1.17	(1.13, 1.2)
ICE WB+INC (CT)	1.26	(1.24, 1.29)	1.08	(1.01, 1.16)	1.21	(1.17, 1.25)
ICE WPC+INC (CT)	1.3	(1.27, 1.33)	1.22	(1.14, 1.32)	1.17	(1.13, 1.2)
FDep (CBG)	1.27	(1.25, 1.3)	1.22	(1.14, 1.3)	1.19	(1.16, 1.23)
FDep (CT)	1.29	(1.27, 1.32)	1.2	(1.12, 1.28)	1.2	(1.16, 1.24)
CRE (CT)	1.25	(1.23, 1.28)	1.2	(1.13, 1.28)	1.15	(1.11, 1.19)
Education (ACS)	1.29	(1.26, 1.32)	1.41	(1.3, 1.54)	1.2	(1.16, 1.25)
Poverty index (ACS)	1.1	(1.08, 1.13)	1.07	(1, 1.14)	1.08	(1.05, 1.12)
Occupation (ACS)	1.14	(1.11, 1.16)	1.11	(1.04, 1.19)	1.12	(1.09, 1.16)

Mortality						
	Not Hispanic		Hispanic		Missing	
	B	95% CI	B	95% CI	B	95% CI
SDI (CBG)	1.15	(1.14, 1.17)	1.13	(1.06, 1.2)	1.12	(1.09, 1.14)
SDI (CT)	1.16	(1.15, 1.18)	1.16	(1.09, 1.23)	1.13	(1.1, 1.15)
SVI (CBG)	1.13	(1.11, 1.15)	1.06	(0.99, 1.13)	1.08	(1.05, 1.1)
SVI (CT)	1.13	(1.12, 1.15)	1.11	(1.04, 1.19)	1.07	(1.05, 1.1)
ADI-GS (CBG)	1.21	(1.19, 1.23)	1.19	(1.12, 1.26)	1.2	(1.17, 1.23)
ADI-GS (CT)	1.21	(1.19, 1.23)	1.19	(1.12, 1.27)	1.19	(1.16, 1.22)
ADI-UW (CBG)	1.21	(1.19, 1.22)	1.16	(1.1, 1.23)	1.25	(1.22, 1.28)
ADI-UW (CT)	1.2	(1.18, 1.22)	1.17	(1.1, 1.23)	1.23	(1.2, 1.26)
NSS7 (CBG)	1.16	(1.15, 1.18)	1.16	(1.09, 1.24)	1.15	(1.12, 1.18)
NSS7 (CT)	1.17	(1.15, 1.19)	1.19	(1.12, 1.27)	1.15	(1.12, 1.18)
ICE WB+INC (CT)	1.17	(1.16, 1.19)	1	(0.93, 1.06)	1.2	(1.17, 1.23)
ICE WPC+INC (CT)	1.18	(1.16, 1.2)	1.16	(1.08, 1.23)	1.14	(1.12, 1.17)
FDep (CBG)	1.2	(1.18, 1.22)	1.14	(1.07, 1.21)	1.17	(1.15, 1.2)
FDep (CT)	1.2	(1.19, 1.22)	1.17	(1.1, 1.24)	1.16	(1.14, 1.19)
CRE (CT)	1.17	(1.15, 1.19)	1.2	(1.13, 1.28)	1.19	(1.16, 1.22)
Education (ACS)	1.45	(1.42, 1.48)	1.4	(1.29, 1.51)	1.39	(1.35, 1.44)
Poverty index (ACS)	1.2	(1.18, 1.22)	1.14	(1.08, 1.21)	1.17	(1.14, 1.2)
Occupation (ACS)	1.22	(1.2, 1.24)	1.22	(1.14, 1.3)	1.2	(1.17, 1.24)

Source: American Family Cohort (AFC) data 2019-2021; American Community Survey (2005-2022); Census Numident (Q3, 2023). Notes: OR = odds ratios; CI = confidence intervals; CBG = census block group; CT = census tract; ACS = American Community Survey. The Census Bureau has reviewed this data product to ensure appropriate access, use, and disclosure avoidance protection of the confidential source data used to produce this product (Data Management System (DMS) number: P-7532672, Disclosure Review Board (DRB) approval number: CBDRB-FY24-POP001-0090).

eTable 7. Coefficients Plotted in Figure 5 (Rurality)

	Hypertension							
	Metropolitan		Micropolitan		Small town		Rural	
	B	95% CI	B	95% CI	B	95% CI	B	95% CI
SDI (CBG)	1.08	(1.07, 1.09)	1.05	(1.03, 1.06)	1.03	(1.01, 1.06)	1.05	(1.02, 1.09)
SDI (CT)	1.09	(1.09, 1.1)	1.05	(1.03, 1.07)	1.04	(1.02, 1.06)	1.11	(1.08, 1.15)
SVI (CBG)	1.05	(1.04, 1.05)	1.04	(1.02, 1.05)	1.07	(1.05, 1.1)	1.02	(0.99, 1.05)
SVI (CT)	1.05	(1.05, 1.06)	1.04	(1.02, 1.06)	1.08	(1.05, 1.11)	1.09	(1.06, 1.12)
ADI-GS (CBG)	1.19	(1.19, 1.2)	1.1	(1.08, 1.12)	1.1	(1.07, 1.13)	1.14	(1.1, 1.18)
ADI-GS (CT)	1.2	(1.19, 1.21)	1.1	(1.08, 1.12)	1.12	(1.08, 1.15)	1.14	(1.1, 1.19)
ADI-UW (CBG)	1.26	(1.25, 1.27)	1.1	(1.08, 1.11)	1.07	(1.04, 1.1)	1.06	(1.02, 1.09)
ADI-UW (CT)	1.26	(1.25, 1.27)	1.09	(1.07, 1.11)	1.06	(1.03, 1.09)	1.04	(1, 1.07)
NSS7 (CBG)	1.13	(1.12, 1.13)	1.06	(1.04, 1.08)	1.04	(1.02, 1.06)	1.08	(1.05, 1.11)
NSS7 (CT)	1.14	(1.13, 1.15)	1.06	(1.05, 1.08)	1.06	(1.04, 1.09)	1.1	(1.07, 1.14)
ICE wb-inc (CT)	1.19	(1.18, 1.19)	1.12	(1.11, 1.14)	1.04	(1.02, 1.07)	1.12	(1.08, 1.15)
ICE wpc-inc (CT)	1.14	(1.14, 1.15)	1.09	(1.07, 1.11)	1.14	(1.11, 1.17)	1.12	(1.09, 1.16)
FDep (CBG)	1.16	(1.16, 1.17)	1.09	(1.07, 1.11)	1.1	(1.08, 1.13)	1.1	(1.07, 1.14)
FDep (CT)	1.17	(1.16, 1.18)	1.11	(1.09, 1.13)	1.11	(1.08, 1.14)	1.11	(1.07, 1.15)
CRE (CT)	1.14	(1.13, 1.15)	1.16	(1.14, 1.18)	1.1	(1.08, 1.13)	1.04	(1.01, 1.07)
Education (ACS)	1.21	(1.2, 1.22)	1.2	(1.17, 1.22)	1.2	(1.17, 1.23)	1.18	(1.15, 1.22)
Poverty index (ACS)	1.03	(1.02, 1.04)	0.98	(0.97, 1)	0.98	(0.96, 1)	0.99	(0.97, 1.01)
Occupation (ACS)	1.1	(1.09, 1.11)	1.08	(1.06, 1.09)	1.06	(1.04, 1.08)	1.07	(1.04, 1.1)

	Diabetes							
	Metropolitan		Micropolitan		Small town		Rural	
	B	95% CI	B	95% CI	B	95% CI	B	95% CI
SDI (CBG)	1.16	(1.15, 1.17)	1.12	(1.09, 1.14)	1.07	(1.04, 1.11)	1.08	(1.04, 1.13)
SDI (CT)	1.18	(1.17, 1.19)	1.11	(1.08, 1.13)	1.11	(1.07, 1.15)	1.1	(1.05, 1.15)
SVI (CBG)	1.13	(1.12, 1.14)	1.11	(1.09, 1.14)	1.1	(1.06, 1.14)	1.09	(1.04, 1.14)
SVI (CT)	1.14	(1.13, 1.15)	1.12	(1.09, 1.15)	1.11	(1.07, 1.15)	1.11	(1.06, 1.15)
ADI-GS (CBG)	1.28	(1.26, 1.29)	1.17	(1.14, 1.2)	1.13	(1.09, 1.18)	1.14	(1.08, 1.2)
ADI-GS (CT)	1.28	(1.27, 1.29)	1.18	(1.15, 1.21)	1.16	(1.11, 1.21)	1.13	(1.07, 1.19)

ADI-UW (CBG)	1.31	(1.3, 1.32)	1.15	(1.13, 1.18)	1.14	(1.1, 1.19)	1.06	(1.01, 1.11)
ADI-UW (CT)	1.31	(1.3, 1.32)	1.15	(1.12, 1.17)	1.11	(1.06, 1.15)	1.05	(1, 1.1)
NSS7 (CBG)	1.22	(1.2, 1.23)	1.13	(1.1, 1.16)	1.08	(1.05, 1.11)	1.12	(1.08, 1.17)
NSS7 (CT)	1.23	(1.22, 1.24)	1.13	(1.1, 1.16)	1.13	(1.09, 1.17)	1.11	(1.07, 1.16)
ICE wb-inc (CT)	1.22	(1.2, 1.23)	1.12	(1.09, 1.15)	1.14	(1.1, 1.18)	1.13	(1.07, 1.18)
ICE wpc-inc (CT)	1.24	(1.23, 1.25)	1.15	(1.12, 1.18)	1.15	(1.11, 1.19)	1.13	(1.07, 1.18)
FDep (CBG)	1.26	(1.25, 1.27)	1.17	(1.14, 1.2)	1.12	(1.08, 1.17)	1.13	(1.08, 1.18)
FDep (CT)	1.26	(1.25, 1.27)	1.19	(1.16, 1.22)	1.16	(1.11, 1.21)	1.15	(1.09, 1.21)
CRE (CT)	1.17	(1.16, 1.18)	1.14	(1.11, 1.16)	1.17	(1.13, 1.21)	1.06	(1.02, 1.1)
Education (ACS)	1.3	(1.28, 1.31)	1.24	(1.21, 1.28)	1.27	(1.23, 1.32)	1.22	(1.16, 1.27)
Poverty index (ACS)	1.11	(1.1, 1.12)	1.05	(1.03, 1.07)	1.04	(1.01, 1.07)	1.02	(0.99, 1.06)
Occupation (ACS)	1.18	(1.16, 1.19)	1.13	(1.1, 1.15)	1.1	(1.07, 1.14)	1.13	(1.09, 1.17)

Chronic Kidney Disease

	Metropolitan		Micropolitan		Small town		Rural	
	B	95% CI	B	95% CI	B	95% CI	B	95% CI
SDI (CBG)	1.19	(1.17, 1.22)	1.04	(1, 1.09)	1.04	(0.97, 1.11)	1.08	(0.99, 1.17)
SDI (CT)	1.21	(1.19, 1.23)	1.05	(1.01, 1.1)	1.02	(0.95, 1.09)	1.14	(1.04, 1.24)
SVI (CBG)	1.13	(1.11, 1.15)	1.03	(0.99, 1.08)	1.04	(0.97, 1.12)	1.14	(1.04, 1.24)
SVI (CT)	1.13	(1.11, 1.15)	0.99	(0.95, 1.04)	1.01	(0.94, 1.09)	1.25	(1.15, 1.36)
ADI-GS (CBG)	1.32	(1.3, 1.35)	1.21	(1.15, 1.27)	1.08	(1, 1.17)	1.15	(1.03, 1.27)
ADI-GS (CT)	1.33	(1.3, 1.35)	1.18	(1.12, 1.25)	1.11	(1.02, 1.21)	1.21	(1.09, 1.35)
ADI-UW (CBG)	1.36	(1.34, 1.39)	1.18	(1.12, 1.24)	1.01	(0.93, 1.09)	0.97	(0.89, 1.07)
ADI-UW (CT)	1.37	(1.35, 1.4)	1.17	(1.11, 1.23)	1	(0.92, 1.08)	0.86	(0.79, 0.94)
NSS7 (CBG)	1.26	(1.24, 1.28)	1.1	(1.05, 1.15)	1.05	(0.98, 1.12)	1.09	(1.01, 1.19)
NSS7 (CT)	1.27	(1.24, 1.29)	1.06	(1.01, 1.11)	1.06	(0.98, 1.13)	1.15	(1.06, 1.24)
ICE wb-inc (CT)	1.28	(1.26, 1.31)	1.22	(1.16, 1.28)	1.15	(1.07, 1.24)	1.1	(0.99, 1.21)
ICE wpc-inc (CT)	1.28	(1.26, 1.3)	1.1	(1.05, 1.15)	1.05	(0.98, 1.13)	1.26	(1.15, 1.39)
FDep (CBG)	1.28	(1.26, 1.31)	1.2	(1.14, 1.26)	1.06	(0.98, 1.15)	1.23	(1.11, 1.35)
FDep (CT)	1.3	(1.28, 1.32)	1.21	(1.15, 1.28)	1.06	(0.98, 1.16)	1.31	(1.19, 1.45)

CRE (CT)	1.26	(1.23, 1.28)	1.23	(1.17, 1.29)	1.09	(1.02, 1.17)	1.18	(1.09, 1.29)
Education (ACS)	1.27	(1.24, 1.3)	1.25	(1.19, 1.32)	1.26	(1.17, 1.36)	1.35	(1.23, 1.47)
Poverty index (ACS)	1.12	(1.1, 1.15)	1.07	(1.03, 1.11)	0.99	(0.93, 1.04)	1.03	(0.96, 1.1)
Occupation (ACS)	1.15	(1.13, 1.18)	1.09	(1.04, 1.14)	1.04	(0.98, 1.1)	1.1	(1.03, 1.18)
Mortality								
	Metropolitan		Micropolitan		Small town		Rural	
	B	95% CI	B	95% CI	B	95% CI	B	95% CI
SDI (CBG)	1.12	(1.11, 1.14)	1.08	(1.04, 1.12)	1.13	(1.08, 1.18)	1.1	(1.04, 1.17)
SDI (CT)	1.13	(1.11, 1.14)	1.11	(1.07, 1.15)	1.17	(1.12, 1.23)	1.08	(1.02, 1.15)
SVI (CBG)	1.08	(1.06, 1.1)	1.08	(1.04, 1.12)	1.15	(1.09, 1.21)	1.08	(1.02, 1.15)
SVI (CT)	1.08	(1.07, 1.1)	1.09	(1.05, 1.13)	1.17	(1.11, 1.23)	1.05	(0.99, 1.12)
ADI-GS (CBG)	1.2	(1.18, 1.21)	1.15	(1.11, 1.19)	1.19	(1.12, 1.26)	1.13	(1.05, 1.22)
ADI-GS (CT)	1.19	(1.17, 1.21)	1.16	(1.11, 1.21)	1.19	(1.12, 1.27)	1.07	(0.99, 1.15)
ADI-UW (CBG)	1.22	(1.2, 1.24)	1.19	(1.15, 1.24)	1.19	(1.12, 1.26)	1.13	(1.06, 1.22)
ADI-UW (CT)	1.21	(1.19, 1.23)	1.19	(1.14, 1.24)	1.17	(1.1, 1.24)	1.11	(1.03, 1.18)
NSS7 (CBG)	1.14	(1.13, 1.16)	1.08	(1.05, 1.12)	1.13	(1.08, 1.19)	1.08	(1.02, 1.15)
NSS7 (CT)	1.15	(1.13, 1.16)	1.11	(1.07, 1.15)	1.14	(1.09, 1.2)	1.03	(0.97, 1.09)
ICE wb-inc (CT)	1.17	(1.15, 1.19)	1.16	(1.12, 1.2)	1.09	(1.04, 1.15)	1.06	(0.99, 1.14)
ICE wpc-inc (CT)	1.13	(1.11, 1.15)	1.14	(1.1, 1.19)	1.17	(1.11, 1.23)	1.15	(1.08, 1.23)
FDep (CBG)	1.17	(1.15, 1.19)	1.12	(1.08, 1.17)	1.13	(1.07, 1.2)	1.06	(0.99, 1.13)
FDep (CT)	1.17	(1.15, 1.19)	1.14	(1.09, 1.19)	1.14	(1.08, 1.21)	1.04	(0.97, 1.11)
CRE (CT)	1.16	(1.14, 1.18)	1.15	(1.11, 1.19)	1.14	(1.08, 1.2)	1.04	(0.98, 1.1)
Education (ACS)	1.4	(1.37, 1.42)	1.41	(1.36, 1.47)	1.51	(1.43, 1.6)	1.45	(1.36, 1.54)
Poverty index (ACS)	1.17	(1.15, 1.19)	1.17	(1.13, 1.2)	1.16	(1.12, 1.21)	1.17	(1.11, 1.22)
Occupation (ACS)	1.21	(1.19, 1.23)	1.18	(1.14, 1.22)	1.19	(1.14, 1.24)	1.15	(1.09, 1.21)

Source: American Family Cohort (AFC) data 2019-2021; American Community Survey (2005-2022); Census Numident (Q3, 2023).
Notes: OR = odds ratios; CI = confidence intervals; CBG = census block group; CT = census tract; ACS = American Community Survey. The Census Bureau has reviewed this data product to ensure appropriate access, use, and disclosure avoidance protection of the confidential source data used to produce this product (Data Management System (DMS) number: P-7532672, Disclosure Review Board (DRB) approval number: CBDRB-FY24-POP001-0090).

eTable 8. Odds Ratios and 95% Confidence Intervals for Associations Between Area and Individual Measures of Social Risk with Health Outcomes and Mortality, Stratified by Gender

	Hypertension			
	Male		Female	
	B	95% CI	B	95% CI
SDI (CBG)	1.05	(1.04, 1.06)	1.11	(1.1, 1.12)
SDI (CT)	1.07	(1.06, 1.08)	1.12	(1.11, 1.13)
SVI (CBG)	1.03	(1.02, 1.04)	1.07	(1.06, 1.08)
SVI (CT)	1.03	(1.03, 1.04)	1.08	(1.08, 1.09)
ADI-GS (CBG)	1.15	(1.14, 1.16)	1.19	(1.18, 1.2)
ADI-GS (CT)	1.15	(1.15, 1.16)	1.2	(1.19, 1.21)
ADI-UW (CBG)	1.19	(1.18, 1.2)	1.21	(1.2, 1.22)
ADI-UW (CT)	1.19	(1.18, 1.2)	1.21	(1.2, 1.21)
NSS7 (CBG)	1.09	(1.09, 1.1)	1.14	(1.13, 1.15)
NSS7 (CT)	1.11	(1.1, 1.12)	1.16	(1.15, 1.17)
ICE WB+INC (CT)	1.15	(1.14, 1.16)	1.2	(1.19, 1.2)
ICE WPC+INC (CT)	1.11	(1.1, 1.12)	1.18	(1.17, 1.19)
FDep (CBG)	1.13	(1.12, 1.14)	1.17	(1.16, 1.18)
FDep (CT)	1.14	(1.13, 1.15)	1.19	(1.18, 1.2)
CRE (CT)	1.12	(1.11, 1.13)	1.16	(1.15, 1.16)
Education (ACS)	1.16	(1.15, 1.18)	1.25	(1.24, 1.26)
Poverty index (ACS)	1.01	(1, 1.02)	1.05	(1.04, 1.05)
Occupation (ACS)	1.08	(1.07, 1.09)	1.08	(1.07, 1.09)
	Diabetes			
	Male		Female	
	B	95% CI	B	95% CI
SDI (CBG)	1.12	(1.11, 1.14)	1.19	(1.18, 1.21)
SDI (CT)	1.13	(1.12, 1.15)	1.22	(1.2, 1.23)
SVI (CBG)	1.11	(1.1, 1.12)	1.15	(1.14, 1.17)
SVI (CT)	1.11	(1.1, 1.12)	1.17	(1.16, 1.19)
ADI-GS (CBG)	1.21	(1.19, 1.22)	1.28	(1.26, 1.3)
ADI-GS (CT)	1.21	(1.19, 1.22)	1.29	(1.27, 1.3)
ADI-UW (CBG)	1.21	(1.2, 1.23)	1.28	(1.26, 1.29)
ADI-UW (CT)	1.21	(1.2, 1.23)	1.27	(1.25, 1.28)
NSS7 (CBG)	1.17	(1.16, 1.18)	1.24	(1.22, 1.25)
NSS7 (CT)	1.18	(1.16, 1.19)	1.26	(1.24, 1.27)
ICE WB+INC (CT)	1.17	(1.16, 1.19)	1.23	(1.22, 1.25)
ICE WPC+INC (CT)	1.19	(1.17, 1.2)	1.29	(1.27, 1.3)
FDep (CBG)	1.2	(1.19, 1.21)	1.28	(1.27, 1.3)
FDep (CT)	1.21	(1.19, 1.22)	1.28	(1.27, 1.3)
CRE (CT)	1.14	(1.13, 1.15)	1.19	(1.18, 1.21)
Education (ACS)	1.25	(1.23, 1.27)	1.32	(1.3, 1.34)
Poverty index (ACS)	1.08	(1.07, 1.1)	1.13	(1.12, 1.15)
Occupation (ACS)	1.12	(1.11, 1.14)	1.15	(1.13, 1.16)
	Chronic Kidney Disease			
	Male		Female	
	B	95% CI	B	95% CI
SDI (CBG)	1.12	(1.1, 1.14)	1.21	(1.18, 1.24)
SDI (CT)	1.14	(1.11, 1.16)	1.22	(1.19, 1.25)
SVI (CBG)	1.09	(1.07, 1.12)	1.14	(1.12, 1.17)
SVI (CT)	1.09	(1.07, 1.11)	1.15	(1.12, 1.18)
ADI-GS (CBG)	1.21	(1.18, 1.23)	1.33	(1.29, 1.36)
ADI-GS (CT)	1.21	(1.18, 1.24)	1.32	(1.29, 1.36)
ADI-UW (CBG)	1.21	(1.18, 1.23)	1.31	(1.28, 1.34)

ADI-UW (CT)	1.2	(1.18, 1.23)	1.3	(1.27, 1.34)
NSS7 (CBG)	1.17	(1.15, 1.2)	1.27	(1.24, 1.3)
NSS7 (CT)	1.18	(1.16, 1.21)	1.27	(1.24, 1.3)
ICE WB+INC (CT)	1.21	(1.19, 1.24)	1.29	(1.26, 1.32)
ICE WPC+INC (CT)	1.21	(1.18, 1.23)	1.3	(1.27, 1.33)
FDep (CBG)	1.2	(1.17, 1.22)	1.3	(1.27, 1.34)
FDep (CT)	1.22	(1.19, 1.24)	1.31	(1.28, 1.34)
CRE (CT)	1.19	(1.16, 1.21)	1.27	(1.24, 1.3)
Education (ACS)	1.19	(1.16, 1.23)	1.36	(1.32, 1.4)
Poverty index (ACS)	1.07	(1.04, 1.09)	1.15	(1.12, 1.17)
Occupation (ACS)	1.08	(1.05, 1.1)	1.14	(1.12, 1.17)
		Mortality		
	Male		Female	
	B	95% CI	B	95% CI
SDI (CBG)	1.12	(1.1, 1.14)	1.15	(1.13, 1.18)
SDI (CT)	1.14	(1.12, 1.16)	1.15	(1.13, 1.17)
SVI (CBG)	1.09	(1.07, 1.1)	1.11	(1.08, 1.13)
SVI (CT)	1.09	(1.07, 1.11)	1.1	(1.08, 1.12)
ADI-GS (CBG)	1.2	(1.18, 1.22)	1.22	(1.19, 1.24)
ADI-GS (CT)	1.2	(1.18, 1.22)	1.2	(1.18, 1.23)
ADI-UW (CBG)	1.23	(1.2, 1.25)	1.23	(1.2, 1.25)
ADI-UW (CT)	1.22	(1.2, 1.24)	1.21	(1.19, 1.24)
NSS7 (CBG)	1.15	(1.13, 1.16)	1.16	(1.14, 1.19)
NSS7 (CT)	1.15	(1.13, 1.17)	1.16	(1.14, 1.19)
ICE WB+INC (CT)	1.18	(1.16, 1.2)	1.2	(1.17, 1.22)
ICE WPC+INC (CT)	1.15	(1.13, 1.17)	1.16	(1.13, 1.18)
FDep (CBG)	1.18	(1.16, 1.2)	1.19	(1.16, 1.21)
FDep (CT)	1.18	(1.16, 1.2)	1.18	(1.15, 1.2)
CRE (CT)	1.18	(1.16, 1.2)	1.17	(1.15, 1.19)
Education (ACS)	1.36	(1.33, 1.39)	1.48	(1.45, 1.52)
Poverty index (ACS)	1.18	(1.16, 1.2)	1.23	(1.21, 1.25)
Occupation (ACS)	1.14	(1.12, 1.16)	1.22	(1.19, 1.24)

Source: American Family Cohort (AFC) data 2019-2021; American Community Survey (2005-2022); Census Numident (Q3, 2023).
Notes: OR = odds ratios; CI = confidence intervals; CBG = census block group; CT = census tract; ACS = American Community Survey. The Census Bureau has reviewed this data product to ensure appropriate access, use, and disclosure avoidance protection of the confidential source data used to produce this product (Data Management System (DMS) number: P-7532672, Disclosure Review Board (DRB) approval number: CBDRB-FY24-POP001-0090).

eTable 9. Odds Ratios and 95% Confidence Intervals for Associations Between Area and Individual Measures of Social Risk with Health Outcomes and Mortality, Stratified by Age

			Hypertension			
	25-44		45-64		>=65	
	OR	95% CI	OR	95% CI	OR	95% CI
SDI (CBG)	1.07	(1.05, 1.09)	1.11	(1.1, 1.12)	1.09	(1.08, 1.1)
SDI (CT)	1.09	(1.07, 1.11)	1.12	(1.11, 1.13)	1.1	(1.09, 1.11)
SVI (CBG)	1.06	(1.04, 1.08)	1.07	(1.06, 1.08)	1.06	(1.05, 1.07)
SVI (CT)	1.06	(1.04, 1.08)	1.08	(1.07, 1.09)	1.07	(1.06, 1.08)
ADI-GS (CBG)	1.19	(1.17, 1.22)	1.19	(1.18, 1.2)	1.14	(1.13, 1.15)
ADI-GS (CT)	1.2	(1.17, 1.22)	1.2	(1.19, 1.21)	1.15	(1.14, 1.16)
ADI-UW (CBG)	1.26	(1.23, 1.28)	1.22	(1.21, 1.23)	1.15	(1.14, 1.16)
ADI-UW (CT)	1.26	(1.24, 1.28)	1.22	(1.21, 1.23)	1.15	(1.14, 1.16)
NSS7 (CBG)	1.13	(1.11, 1.15)	1.15	(1.14, 1.16)	1.11	(1.1, 1.12)
NSS7 (CT)	1.14	(1.12, 1.16)	1.16	(1.15, 1.17)	1.13	(1.11, 1.14)
ICE wb-inc (CT)	1.2	(1.18, 1.22)	1.19	(1.18, 1.2)	1.14	(1.13, 1.15)
ICE wpc-inc (CT)	1.17	(1.14, 1.19)	1.17	(1.16, 1.18)	1.14	(1.13, 1.15)
FDep (CBG)	1.18	(1.15, 1.2)	1.18	(1.17, 1.19)	1.13	(1.12, 1.14)
FDep (CT)	1.17	(1.15, 1.19)	1.19	(1.18, 1.2)	1.14	(1.13, 1.15)
CRE (CT)	1.13	(1.11, 1.15)	1.14	(1.13, 1.15)	1.09	(1.08, 1.1)
Education (ACS)	1.26	(1.23, 1.29)	1.14	(1.13, 1.15)	1.11	(1.09, 1.12)
Poverty index (ACS)	1.07	(1.05, 1.09)	1.07	(1.06, 1.08)	1.08	(1.07, 1.1)
Occupation (ACS)	1.12	(1.1, 1.14)	1.11	(1.1, 1.12)	1.06	(1.05, 1.07)
			Diabetes			
	25-44		45-64		>=65	
	OR	95% CI	OR	95% CI	OR	95% CI
SDI (CBG)	1.22	(1.18, 1.26)	1.19	(1.18, 1.21)	1.14	(1.13, 1.15)
SDI (CT)	1.24	(1.2, 1.28)	1.21	(1.19, 1.22)	1.15	(1.13, 1.16)
SVI (CBG)	1.19	(1.15, 1.23)	1.16	(1.15, 1.18)	1.12	(1.1, 1.13)
SVI (CT)	1.2	(1.17, 1.24)	1.17	(1.16, 1.18)	1.13	(1.12, 1.14)
ADI-GS (CBG)	1.32	(1.28, 1.36)	1.26	(1.24, 1.27)	1.19	(1.18, 1.21)
ADI-GS (CT)	1.32	(1.28, 1.36)	1.27	(1.25, 1.28)	1.19	(1.17, 1.2)
ADI-UW (CBG)	1.3	(1.26, 1.34)	1.26	(1.24, 1.27)	1.2	(1.18, 1.21)
ADI-UW (CT)	1.3	(1.26, 1.34)	1.25	(1.24, 1.27)	1.19	(1.18, 1.2)
NSS7 (CBG)	1.28	(1.24, 1.32)	1.23	(1.22, 1.25)	1.17	(1.16, 1.19)
NSS7 (CT)	1.29	(1.25, 1.33)	1.25	(1.23, 1.26)	1.17	(1.16, 1.19)
ICE wb-inc (CT)	1.25	(1.21, 1.29)	1.21	(1.2, 1.23)	1.16	(1.14, 1.17)
ICE wpc-inc (CT)	1.33	(1.29, 1.37)	1.27	(1.25, 1.28)	1.19	(1.17, 1.2)
FDep (CBG)	1.32	(1.28, 1.36)	1.26	(1.25, 1.28)	1.19	(1.18, 1.21)
FDep (CT)	1.31	(1.27, 1.36)	1.27	(1.25, 1.28)	1.2	(1.18, 1.21)
CRE (CT)	1.22	(1.18, 1.25)	1.17	(1.16, 1.19)	1.1	(1.09, 1.11)
Education (ACS)	1.39	(1.33, 1.45)	1.23	(1.21, 1.24)	1.17	(1.16, 1.19)
Poverty index (ACS)	1.17	(1.14, 1.21)	1.16	(1.15, 1.18)	1.12	(1.11, 1.14)
Occupation (ACS)	1.2	(1.16, 1.24)	1.18	(1.16, 1.19)	1.13	(1.12, 1.15)
			Chronic Kidney Disease			
		25-44	45-64	45-64	>=65	>=65
	OR	95% CI	OR	95% CI	OR	95% CI
SDI (CBG)	1.18	(1.07, 1.3)	1.22	(1.18, 1.25)	1.16	(1.14, 1.18)
SDI (CT)	1.15	(1.05, 1.27)	1.24	(1.2, 1.28)	1.17	(1.15, 1.19)
SVI (CBG)	1.13	(1.03, 1.25)	1.15	(1.11, 1.18)	1.13	(1.11, 1.15)
SVI (CT)	1.17	(1.06, 1.28)	1.15	(1.12, 1.18)	1.13	(1.11, 1.15)
ADI-GS (CBG)	1.2	(1.09, 1.33)	1.29	(1.25, 1.33)	1.23	(1.21, 1.25)
ADI-GS (CT)	1.23	(1.11, 1.35)	1.29	(1.25, 1.33)	1.23	(1.2, 1.25)
ADI-UW (CBG)	1.12	(1.02, 1.23)	1.29	(1.25, 1.33)	1.22	(1.19, 1.24)

ADI-UW (CT)	1.13	(1.03, 1.24)	1.28	(1.24, 1.32)	1.21	(1.19, 1.24)
NSS7 (CBG)	1.22	(1.11, 1.35)	1.25	(1.22, 1.29)	1.21	(1.19, 1.23)
NSS7 (CT)	1.24	(1.13, 1.37)	1.26	(1.22, 1.3)	1.21	(1.18, 1.23)
ICE wb-inc (CT)	1.19	(1.08, 1.31)	1.28	(1.24, 1.32)	1.21	(1.18, 1.23)
ICE wpc-inc (CT)	1.29	(1.17, 1.42)	1.29	(1.25, 1.33)	1.23	(1.21, 1.26)
FDep (CBG)	1.24	(1.12, 1.37)	1.28	(1.24, 1.32)	1.23	(1.2, 1.25)
FDep (CT)	1.22	(1.11, 1.35)	1.28	(1.24, 1.32)	1.24	(1.22, 1.27)
CRE (CT)	1.21	(1.1, 1.33)	1.23	(1.2, 1.27)	1.15	(1.13, 1.18)
Education (ACS)	1.12	(0.98, 1.27)	1.16	(1.12, 1.2)	1.16	(1.14, 1.19)
Poverty index (ACS)	1.17	(1.07, 1.29)	1.19	(1.15, 1.22)	1.15	(1.13, 1.17)
Occupation (ACS)	1.18	(1.07, 1.31)	1.13	(1.1, 1.17)	1.11	(1.09, 1.13)
Mortality						
	25-44		45-64		>=65	
	OR	95% CI	OR	95% CI	OR	95% CI
SDI (CBG)	1.14	(1.06, 1.22)	1.23	(1.2, 1.26)	1.12	(1.11, 1.14)
SDI (CT)	1.17	(1.09, 1.25)	1.23	(1.2, 1.26)	1.13	(1.11, 1.14)
SVI (CBG)	1.13	(1.06, 1.21)	1.15	(1.12, 1.18)	1.1	(1.08, 1.12)
SVI (CT)	1.12	(1.05, 1.2)	1.16	(1.13, 1.19)	1.09	(1.08, 1.11)
ADI-GS (CBG)	1.25	(1.17, 1.34)	1.29	(1.26, 1.33)	1.16	(1.14, 1.18)
ADI-GS (CT)	1.25	(1.17, 1.34)	1.28	(1.25, 1.31)	1.15	(1.13, 1.16)
ADI-UW (CBG)	1.26	(1.18, 1.35)	1.28	(1.24, 1.31)	1.18	(1.16, 1.2)
ADI-UW (CT)	1.25	(1.17, 1.33)	1.26	(1.23, 1.3)	1.17	(1.15, 1.19)
NSS7 (CBG)	1.21	(1.13, 1.3)	1.25	(1.22, 1.28)	1.13	(1.11, 1.14)
NSS7 (CT)	1.24	(1.16, 1.33)	1.25	(1.22, 1.28)	1.12	(1.11, 1.14)
ICE wb-inc (CT)	1.18	(1.1, 1.26)	1.24	(1.21, 1.27)	1.14	(1.12, 1.16)
ICE wpc-inc (CT)	1.24	(1.15, 1.32)	1.24	(1.2, 1.27)	1.12	(1.1, 1.14)
FDep (CBG)	1.25	(1.17, 1.34)	1.27	(1.24, 1.3)	1.14	(1.12, 1.16)
FDep (CT)	1.27	(1.19, 1.36)	1.26	(1.23, 1.29)	1.14	(1.12, 1.15)
CRE (CT)	1.22	(1.14, 1.3)	1.2	(1.17, 1.23)	1.1	(1.08, 1.12)
Education (ACS)	1.54	(1.41, 1.68)	1.31	(1.27, 1.35)	1.29	(1.26, 1.31)
Poverty index (ACS)	1.33	(1.25, 1.42)	1.3	(1.26, 1.33)	1.25	(1.23, 1.27)
Occupation (ACS)	1.32	(1.23, 1.42)	1.25	(1.22, 1.29)	1.17	(1.15, 1.19)

Source: American Family Cohort (AFC) data 2019-2021; American Community Survey (2005-2022); Census Numident (Q3, 2023).
Notes: OR = odds ratios; CI = confidence intervals; CBG = census block group; CT = census tract; ACS = American Community Survey. The Census Bureau has reviewed this data product to ensure appropriate access, use, and disclosure avoidance protection of the confidential source data used to produce this product (Data Management System (DMS) number: P-7532672, Disclosure Review Board (DRB) approval number: CBDRB-FY24-POP001-0090).

eFigure. Odds Ratios and 95% Confidence Intervals for Associations Between Area Measures (Deciles) of Social Risk with Health Outcomes and Mortality

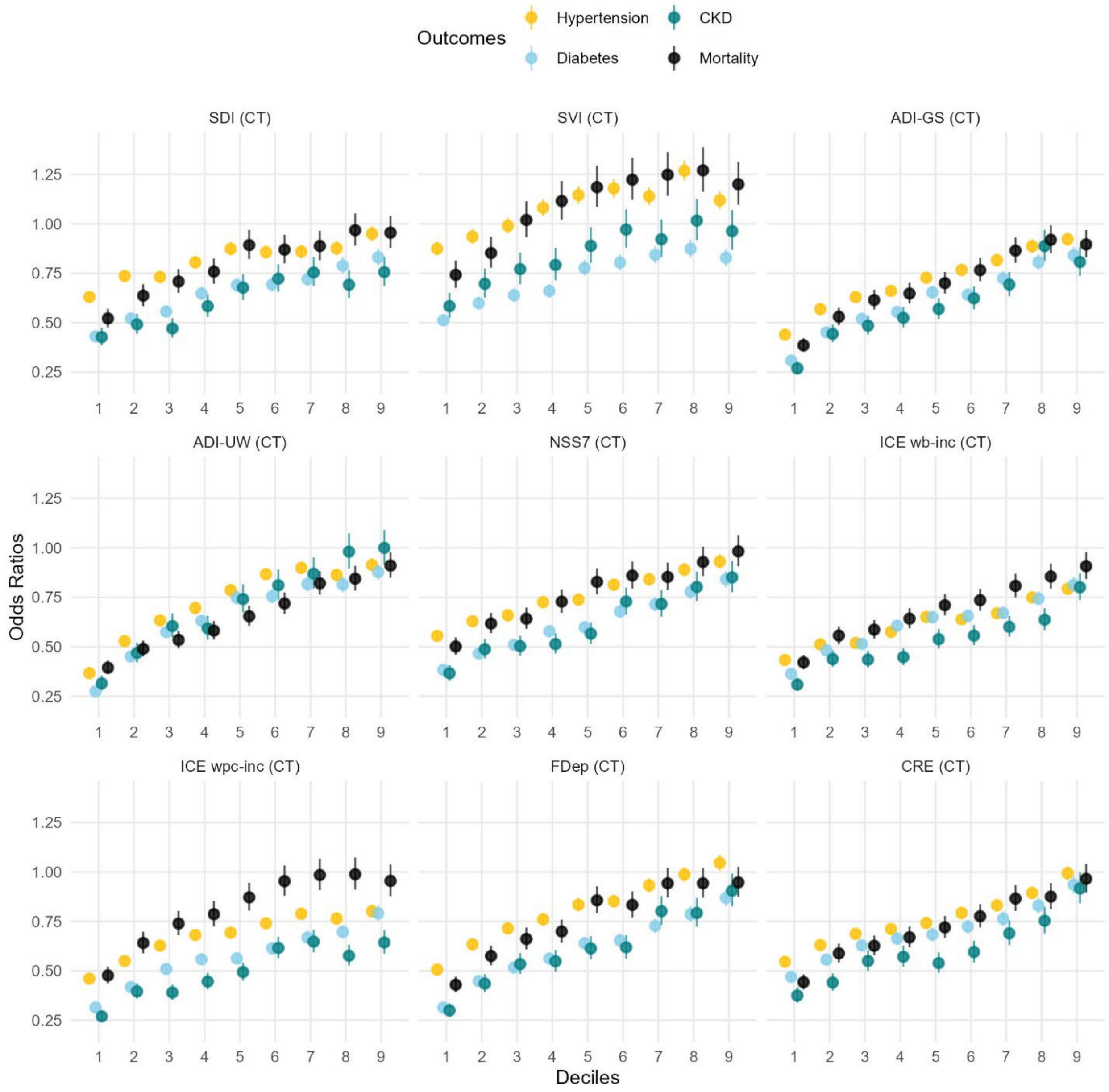

Source: American Family Cohort (AFC) data 2019-2021; American Community Survey (2005-2022); Census Numident (Q3, 2023). Notes: Models are unadjusted; CKD = chronic kidney disease; CT = census tract. The Census Bureau has reviewed this data product to ensure appropriate access, use, and disclosure avoidance protection of the confidential source data used to produce this product (Data Management System (DMS) number: P-7532672, Disclosure Review Board (DRB) approval number: CBDRB-FY25-POP001-0068).
