# Supplementary material for: Validating 8 Area-Based Measures of Social Risk for Predicting Health and Mortality
Source: JAMA Health Forum. 2025 Aug 15;6(8):e252669. doi: 10.1001/jamahealthforum.2025.2669 (PMC12357199; doi:10.1001/jamahealthforum.2025.2669)
Supplement: Supplement 2. — Data Sharing Statement [file jamahealthforum-e252669-s002.pdf]

Data Sharing Statement

Limburg. Validating 8 Area-Based Measures of Social Risk for Predicting Health and Mortality. *JAMA Health Forum*. Published August 15, 2025. doi:10.1001/jamahealthforum.2025.2669

Data
Data available: No

Additional Information
Explanation for why data not available: The linked data underlying this article cannot be shared publicly as they are protected by Title 13 of the U.S. Code which authorizes the Census Bureau to collect information from other entities and requires the Census Bureau to keep the information confidential and use it only for statistical purposes. We will, however, make all of the data for the area based measures of social risk available, including the measures and the code to create the measures.
